# How short is too short for amyloid fibrils?: Molecular dynamics of oligomers of infectious prion core structures

**DOI:** 10.1016/j.jbc.2025.110390

**Published:** 2025-06-19

**Authors:** Efrosini Artikis, Amitava Roy, Byron Caughey

**Affiliations:** 1 Laboratory of Neurological Infections and Immunity, Rocky Mountain Laboratories, Division of Intramural Research, National Institute of Allergy and Infectious Diseases, National Institutes of Health, Hamilton, Montana, USA; 2 Bioinformatics and Computational Biosciences Branch, Rocky Mountain Laboratories, Division of Intramural Research, National Institute of Allergy and Infectious Diseases, National Institutes of Health, Hamilton, Montana, USA

**Keywords:** prion, prion disease, amyloid, oligomer, protein stability

## Abstract

In many proteinopathies, the relative conformations of amyloid fibrils *versus* smaller oligomers remain unclear. Most tissue-derived isolates of infectious prion protein (PrP) prions are predominantly fibrillar. A few studies have asserted that prion amyloid fibrils efficiently disassemble into dimeric to tetrameric “elemental bricks” under certain detergent or chaotropic conditions, but our companion paper provides strong evidence to the contrary. Given our difficulties in isolating detectable amounts of small oligomeric (2-4-mer) prions, we performed molecular dynamics simulations to test the abilities of small fragments (dimers to 25-mers) of cryo-EM-based infectious prion fibril core structures to retain their conformational integrity. We show that dimers of the aRML prion structure lost most of their original secondary and tertiary structure within <<1 μs, while trimers maintained some intermolecular **β**-sheets. Further increases in fragment size helped preserve major structural motifs and the integrity of the templating surfaces responsible for self-propagation. In simulations of octamers and/or 25-mers, even at elevated temperatures, no fragmentation was observed for aRML, 22L, or 263K prion strains, although the terminal chains were substantially destabilized. Together, our results provide evidence that oligomeric fragments of prion fibril cores as small as tetramers retain substantial structural integrity. Our findings suggest that, as exemplified by PrP fibrils, short cores as small as tetramers may be stable enough to account for bioactive oligomeric species detected in brain extracts from patients with amyloidoses. However, the lack of observed spontaneous core fragmentation suggests that prion oligomers might be rare *in vivo* and/or produced by non-autonomous physiological cleavage processes.

In multiple illnesses such as Alzheimer’s, Parkinson’s and prion diseases, specific proteins form amyloid fibrils that can often be observed histologically as amyloid plaques. The accumulation of such amyloid fibrils has been hypothesized to be toxic and a primary cause of amyloidoses (1, 2). On the other hand, in some disorders in which amyloid plaques are found, plaque burden does not appear to correlate well with clinical severity (1, 3). Such findings, as well as the detection of various apparently non-fibrillar oligomeric protein assemblies in diseased tissue, have supported hypotheses that such oligomers are more pathogenic than larger amyloid fibrils. However, although the high-resolution structures of many *ex vivo* disease-associated amyloid fibrils have been solved (4), empirically based structures of smaller oligomers remain elusive. Without knowledge of the latter, it remains possible that many of the oligomers that have been described share the β-sheet core structure of larger amyloid fibrils but are too short to have elongated fibrillar morphologies. This raises the question: How short can pathological amyloid structures be before they lose their structural integrity and prion-like, strain-specific-self-propagating activities?

We approached this issue using prion protein (PrP) prions as highly infectious examples of pathological amyloids. PrP-based prion diseases are untreatable, lethal, and transmissible neurodegenerative disorders (5). The transmissible agents (prions) of these diseases are composed primarily of misfolded assemblies of the host’s PrP molecules. The normal form of PrP (PrP^C^) has a disordered N-terminal domain and largely α-helical C-terminal domain (6), whereas the infectious (prion) forms (generically called PrP^Sc^) have a high β-sheet content and lack α-helical secondary structure (7, 8, 9, 10, 11, 12, 13, 14, 15, 16). The structures of several highly infectious rodent and deer prion strains have been solved at near-atomic resolution by cryo-electron microscopy (cryo-EM), revealing amyloid fibrils with ordered cores comprised of PrP monomers (*i.e.*, polypeptide chains) aligned along the fibril axis *via* a serpentine series of loops and parallel, in-register intermolecular β-sheets (PIRIBS) (10, 11, 12, 13, 14, 15, 16, 17). Such architectures are common in amyloids and are well suited to explain prion propagation and strain variation (13, 14, 18, 19). The available *ex vivo* rodent prion structures share other general features as well: hairpin-shaped motifs that we have called the N-, middle, and disulfide arches; a steric zipper between the N-terminal residues of the ordered core and the head of the middle arch; and a central, and often staggered, interface between the major N- and C-terminal lobes of the core (Fig. 1). The N-linked glycans and C-terminal glycophosphatidylinositol (GPI) anchor, which are present in most prion strains, project outward from the surface of the fibril core. The detailed conformations of these shared core structural features can differ between prion strains (10, 11, 12, 13, 14, 15, 16), providing conformational templates for growth (15, 16). Such templates have long been postulated to serve as the molecular basis of prion strain differentiation (20, 21, 22).

**Figure 1:**
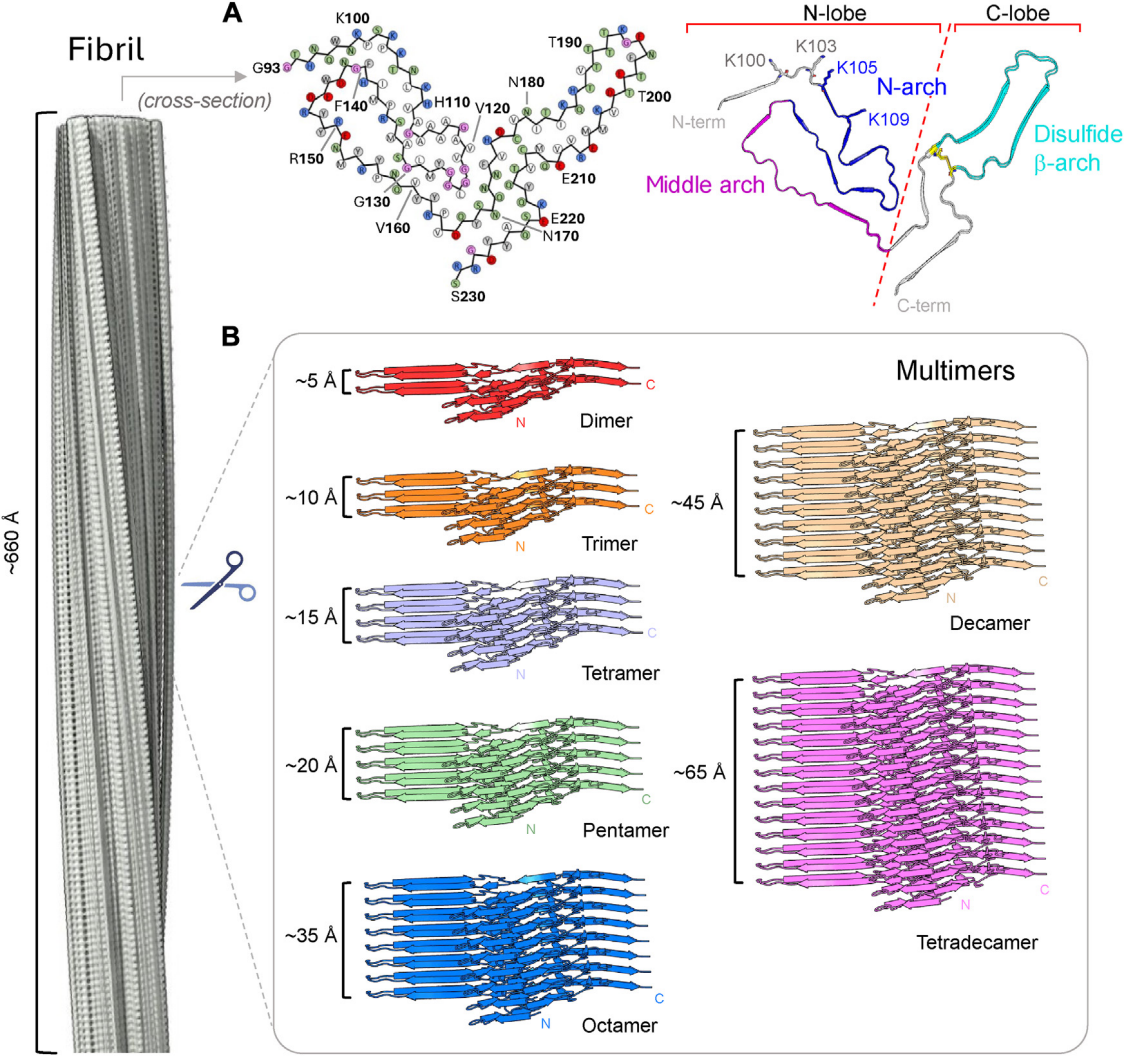
**Oligomeric fragments of aRML fibril (PDB****7TD6****).***A*, cross-section displaying residue orientations in *circles* (*green*, polar; *blue*, basic; *red*, acidic; *white*, aliphatic; *gray*, aromatic; *pink*, glycine). Cross-section on *right* highlights important structural motifs. Lysines 100, 103, 105 and 109 in the N-lobe comprise the central lysine cluster (CLC). *B*, structures of dimer through tetradecamer derived from aRML fibril.

The available cryo-EM-based structures provide static views of the predominant, and most highly ordered, features of infectious prion amyloids. However, to understand how prions grow, replicate, and spread, it is important to consider the dynamics of these structures. One key question is whether the terminal chains in prion fibrils are more mobile than interior chains in a way that might influence their interactions with incoming PrP^C^ molecules. Another uncertainty is the minimum stable size of PrP^Sc^ assemblies. Under sub-denaturing chaotropic conditions, scrapie prion infectivity and the ability to convert PrP^C^ to a PrP^Sc^-like protease-resistant form remain associated with aggregates of >2000 kDa, *i.e.* the mass equivalent of ∼60+ PrP molecules (23, 24). Further studies have shown that sonication in strong detergent can generate a minor subpopulation of smaller prion particles of ∼300 to 600 kDa (mass of ∼14–28 PrP molecules) that are more infectious per unit protein than larger fibrils and that the minimum assembly capable of inducing PrP conversion is larger than a pentamer (25). However, early radiation inactivation studies provided evidence for effective infectivity target sizes of 55 to 150 kDa, *i.e.*, comparable to 2 to 6 PrP molecules (26, 27). Others have reported that PrP^Sc^ units as small as dimers to tetramers can be efficiently generated by spontaneous disassembly under certain conditions while retaining infectivity and/or strain-dependent templating activity (28, 29, 30, 31, 32). However, in our own attempts to replicate the most recent of those studies (33) we found that the appearance of disassembly into small oligomers was primarily due to experimental artifacts. In any case, whether particles on the small end of the range of infectious prion assemblies are short versions of PIRIBS fibrils, or distinct oligomeric architectures, remains to be ascertained. Also unclear is their relative abundance and pathological significance compared to larger amyloid fibrils under the physiological conditions in the brain. Importantly, not only do some smaller prion particles have higher specific infectivity per unit protein (25), but they also appear to spread more readily within the brain (34). Finally, as prion fragmentation has been reported to dominate the kinetics of prion replication *in vivo* (35), it is relevant to evaluate the relative conformational stabilities of prion assemblies of different sizes.

Here, motivated in part by our difficulties in generating detectable amounts of the previously proposed small infectious prion oligomers with 2 to 4 monomers (33), we have started with the known core cryo-electron microscopy-based structure of infectious *ex vivo* prion fibrils from rodent-adapted prion strains and performed molecular dynamics (MD) simulations on assemblies ranging in size from 2 to 25 monomers to assess their relative conformational mobilities *in silico*.

## Results

To assess conformational stabilities and dynamics of PrP^Sc^ fragments *in silico*, we initially built assemblies of various lengths using the cryo-EM structure of brain-derived fibrils of the anchorless RML (aRML) PrP^Sc^ mouse strain (PDB 7TD6) (11). All assemblies had identical starting conformations differing only by the number of chains (Fig. 1). These models represented the ordered core of the aRML prion and lacked the disordered N-terminal domains and post-translational modifications. However, despite the fact that aRML prions lack GPI moieties and are sparsely glycosylated relative to wild-type RML prions (36), their core structure is nearly superimposable on that of the GPI-anchored, glycosylated wild-type PrP^Sc^ (12) (Fig. S1). These factors, as well as the predominant accumulation of aRML prions in extracellular amyloid plaques rather than membrane-bound assemblies, support the *in vivo* relevance of simulations without the inclusion of complex N-linked glycans, GPI anchors, or lipid bilayers.

### Effect of fragment size on maintenance of the PrP^Sc^ structural motifs

**Figure 2:**
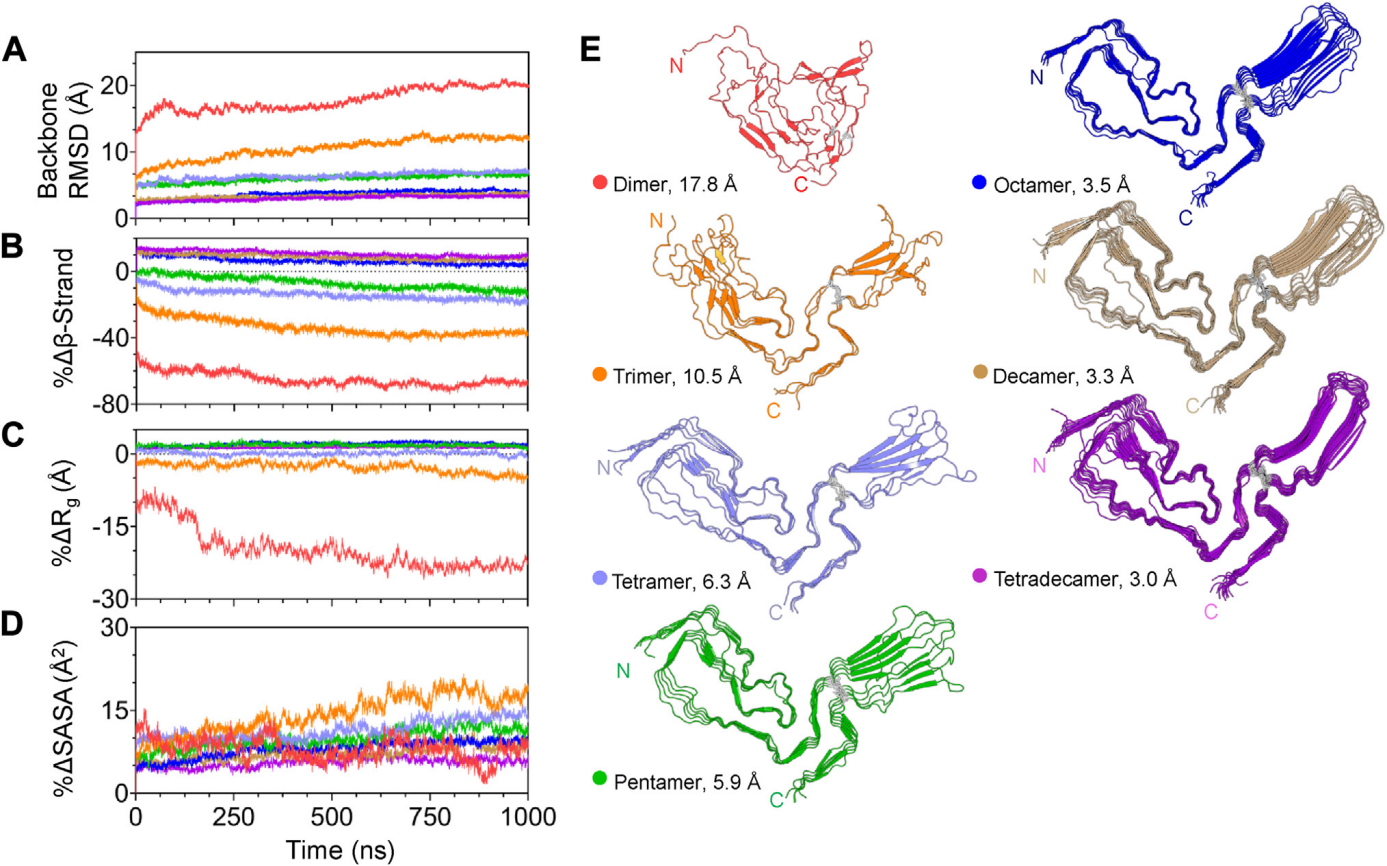
**Molecular dynamics analysesof PrP^Sc^ oligomers.***A*, average backbone RMSD of the last 1 μs of molecular dynamics simulations for each oligomer (as color-coded in E) with respect to the cryo-EM structure plotted against time (ns). *B*, average percent change in total β-sheet content with respect to the cryo-EM structure for the last 1 μs of simulation time. *C*, average percent change in radius of gyration with respect to the cryo-EM structure for the last 1 μs of simulation time. *D*, average percent change of total solvent accessible surface area (SASA) with respect to the cryo-EM structure for the last 1 ms of simulation time. *E*, Last frame of a single simulation for each corresponding fragment. Cross-sections are colored to match the lines in plots (*A–D*).

We performed up to four independent ≥1 μs MD simulations (see Table S1 for details) of each aRML PrP^Sc^ fragment. The convergence of the simulations was assessed by calculating backbone root mean square deviation (RMSD) relative to the starting structures of the production runs (Fig. S2), and all trajectories were analyzed as averages of the last 1 μs of simulation time unless otherwise noted. For comparative purposes between fragments of different lengths, Figure 2*A* depicts the average RMSD trajectory of each fragment relative to the cryo-EM structure.

Dimers and trimers underwent major conformational changes with mean backbone RMSD values of 17.8 Å and 10.5 Å, respectively. There was considerable heterogeneity in the structural ensembles within and between runs of the dimer (Fig. S3). These deviations were largely due to the breakdown of secondary structure, which for the dimer resulted in an average ∼70% reduction in total β-sheet content (Fig. 2*B*) compared to the cryo-EM structure. Consequently, the dimer was predominantly unfolded and contained little of the original secondary structure (Fig. S3). However, short parallel β-strands in the N-lobe formed by residues MET137-HIS139 remained intact for a large portion of the simulation time for all the runs (Fig. S3*B*).

The high solvent-accessible surface area (SASA) of the dimer was largely due to the mostly planar nature of monomers within the PIRIBS architecture, which allowed both protein chains to be solvent-exposed. Early in the simulations, increased hydration of the hydrophobic core in the N-arch led to structural instability. After an initial increase in SASA (Fig. 2*D*), the dimer experienced hydrophobic collapse as suggested by the decrease in radius of gyration (Fig. 2*C*). As the dimer chains became disordered, the overall structure became more compact.

The trimer diverged markedly from the initial conformation but, compared to the dimer, maintained a higher percentage of β-sheet and regions of modest structural stability, confined mainly to the N-arch and C-lobe (Fig. 2*E*). Unfolding of the middle and disulfide arches exposed residues of the middle chain and contributed to an increase in SASA (Fig. 2*D*) and a marginal decrease in the radius of gyration (Fig. 2*C*). Twisting of the N- and C-lobes exposed hydrophobic residues in the middle chain and disrupted the relative planarity of the starting structure, making the trimer slightly more compact (Fig. 2*E*).

The tetramer and pentamer had backbone RMSD values of 6.3 Å and 5.9 Å, respectively, and retained more of their starting conformation than the two smaller species. The PIRIBS architecture was largely maintained with an overall β-sheet reduction of only 10 to 20% (Fig. 2*B*). The converged structures had well-ordered N-lobes, preserving the N-arch and large sections of the middle arch motif. Interestingly, both the tetramer and pentamer displayed a twist in the middle arch, like the trimer, but to a lesser extent (see section below on large domain motion). The major contributors to the high RMSD values were disorder of the terminal chains, splaying in residues 150-160 of the middle arch, and flexibility of the disulfide β-arch. The radius of gyration did not differ noticeably from the original structure despite a small increase in SASA (Fig. 2, *C* and *D*).

The octamers and larger PrP^Sc^ multimers had still greater structural stability with RMSD values of 3.0 Å to 3.5 Å. The additional chains reinforced the PIRIBS architecture and reduced the loss of secondary structure (<10% change in β-sheet content, Fig. 2*B*). These multimers maintained their major structural motifs, radii of gyration, and solvent exposures (Fig. 2, *C* and *D*). The terminal chains displayed disorder in the middle and disulfide β-arches, a common observation among all the oligomers. The relative instability in the disulfide β-arch was apparent despite the rigidity provided by additional chains.

### Conformational dynamics of PrP^Sc^ subunits

Next, we probed the impact of multimer length on conformational dynamics by computing the average Cα root mean square fluctuation (RMSF), which provides a description of individual residue motion. Due to the structural plasticity of the dimer within and between MD runs, RMSF analysis did not provide a meaningful assessment of residue mobility.

**Figure 3:**
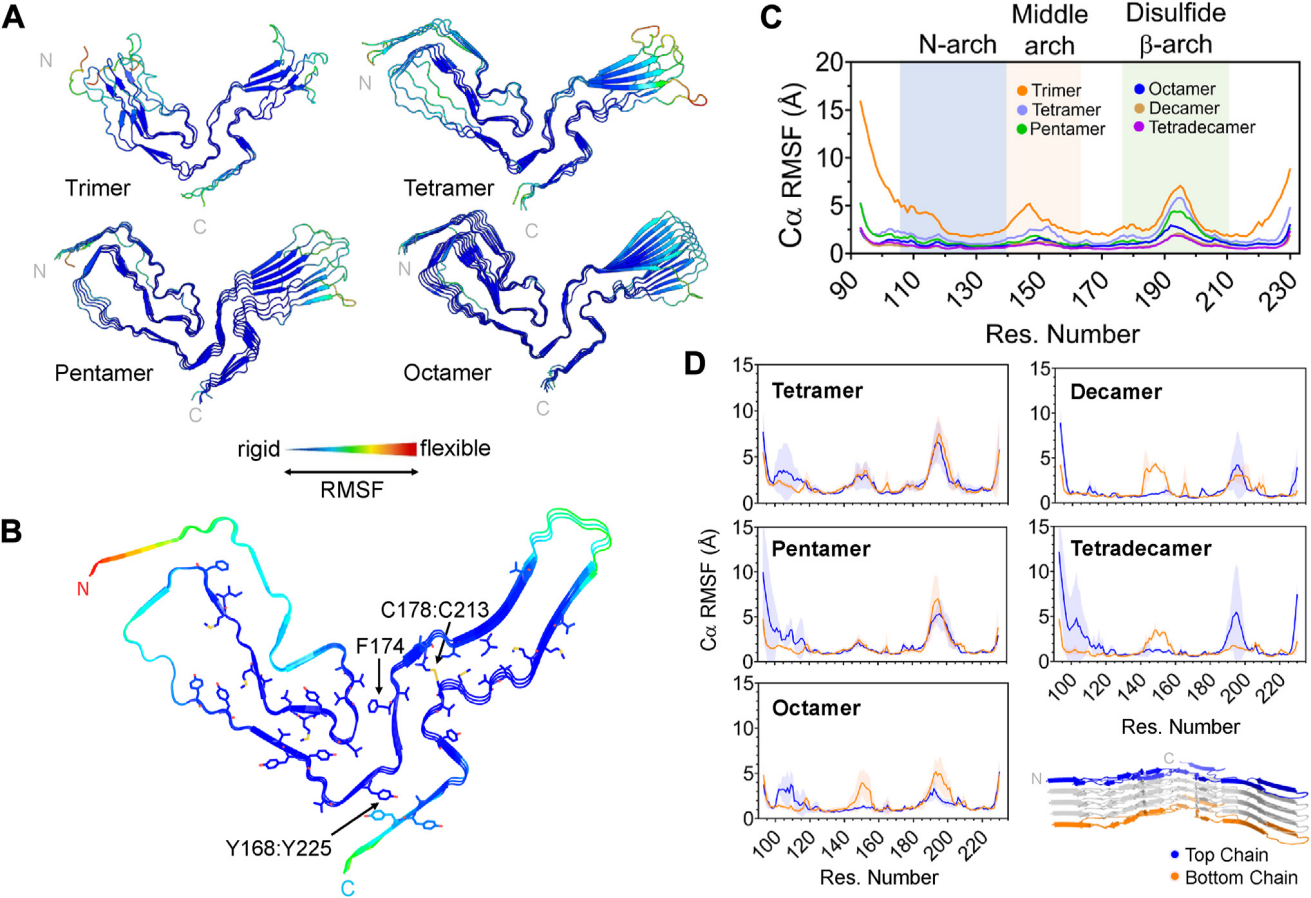
**Fluctuations withinPrP^Sc^ oligomers.***A*, average Cα RMSF values are displayed on the backbone (last frame from one simulation) of each PrP^Sc^ oligomer. The color gradient from *blue* to *red* denotes relatively rigid and flexible regions, respectively. *B*, average Cα RMSF of the trimer projected onto the backbone of the trimer starting structure. The disulfide bond (C178:C213) and the sidechains of hydrophobic residues with low Cα RMSF values are shown in ‘licorice’ representation. Residue F174, and the aromatic interaction formed by Y168:Y225, are marked with arrows. *C*, average Cα RMSF (Å) plotted per residue of the PrP^Sc^ oligomers. Three main structural motifs and their corresponding residues are shaded in *blue*, *orange* and *green* (N-, middle and disulfide β-arches, respectively). *D*, the average Cα RMSF values of the *top* (*blue*) and *bottom* (*orange*) chains of each PrP^Sc^ oligomer (average, line; shadow, standard deviation).

Despite substantial conformational changes in the trimer, regions of reduced fluctuation were identified in the N-arch (residues ∼120-140), stretches of the middle arch (residues ∼160-166) and in the residues preceding the disulfide β-arch (∼168-178) (Figs. 3, *A* and *C*, and S4). Hydrophobic interactions in the N-arch of the trimer contributed to structural rigidity and reduced the motion of neighboring residues in the middle arch (Fig. 3*B*). Additionally, the C-lobe displayed reduced mobility near the disulfide bond (cysteines 178:213) and in the surrounding hydrophobic residues, including a π-π interaction near the C-terminus (tyrosines 168:225).

Similar trends were observed in the tetramer and pentamer simulations, in which regions of relative rigidity extended into the N-proximal steric zipper, the central lysine cluster (CLC; K101, K103, K105, and K109), and the top of the middle arch (Fig. 3, *A* and *C*). In the tetramer, the largest fluctuations occurred toward the tip of the disulfide β-arch (residues ∼190-200) and in the residues near the N- (residues 93-95) and C-termini (227-230). Additionally, the N-lobe of the tetramer fluctuated notably in the solvent-exposed portion of the middle arch (residues 145-155) (Fig. S5, arrow). In the N-lobe of the pentamer, reduced residue mobility reinforced all the structural motifs seen in the starting PrP^Sc^ conformation (N- and middle arches). However, modest flexibility was observed in the interface between the N- and C-lobes (residues 119-120 and 174-176) in both the tetramer and pentamer.

The octamer, decamer, and tetradecamer (Figs. 3, S6 and S7) remained relatively rigid throughout the core, except for major fluctuations at the tip of the disulfide β-arch, the N-and C-termini, and in the terminal chains. Increased fluctuation was seen in the top chain of the tetradecamer near residues 101-105 (Fig. S7), in which residue K103 of the CLC is flanked by P101 and P104. Flipping of the pyrrolidine ring in proline between the endo and exo conformations largely contributed to the observed flexibility due to covalent bonding of the side chain to the protein backbone. (Fig. S7).

Motion in the terminal chains of the PrP^Sc^ assemblies was a major contributor overall to the average Cα RMSF (Fig. 3*C*), with a few notable differences between the top and bottom chains (Fig. 3*D*). For the tetramer and pentamer, average fluctuations in the top chains of the N-lobe were predominantly in the far N-terminus, specifically in the region of the CLC (Fig. 3*D*). In larger species, the top chain indicated fluctuations in the CLC, whereas the bottom chains experienced more motion in the middle arch between residues 140-160 (Fig. 3*D*). This was especially apparent in the terminal chains of the octamer, decamer, and tetradecamer. Both chains displayed high flexibility in the disulfide β-arch in all multimers except for the tetradecamer in which only the top chain exhibited increased fluctuation. These slight size-dependent variations suggest differential plasticity in the multimer poles, likely due to the non-coplanarity of the N- and C-lobes of monomers within the fibril (see Discussion).

#### Large domain motion observed in smaller PrP^Sc^ fragments

**Figure 4:**
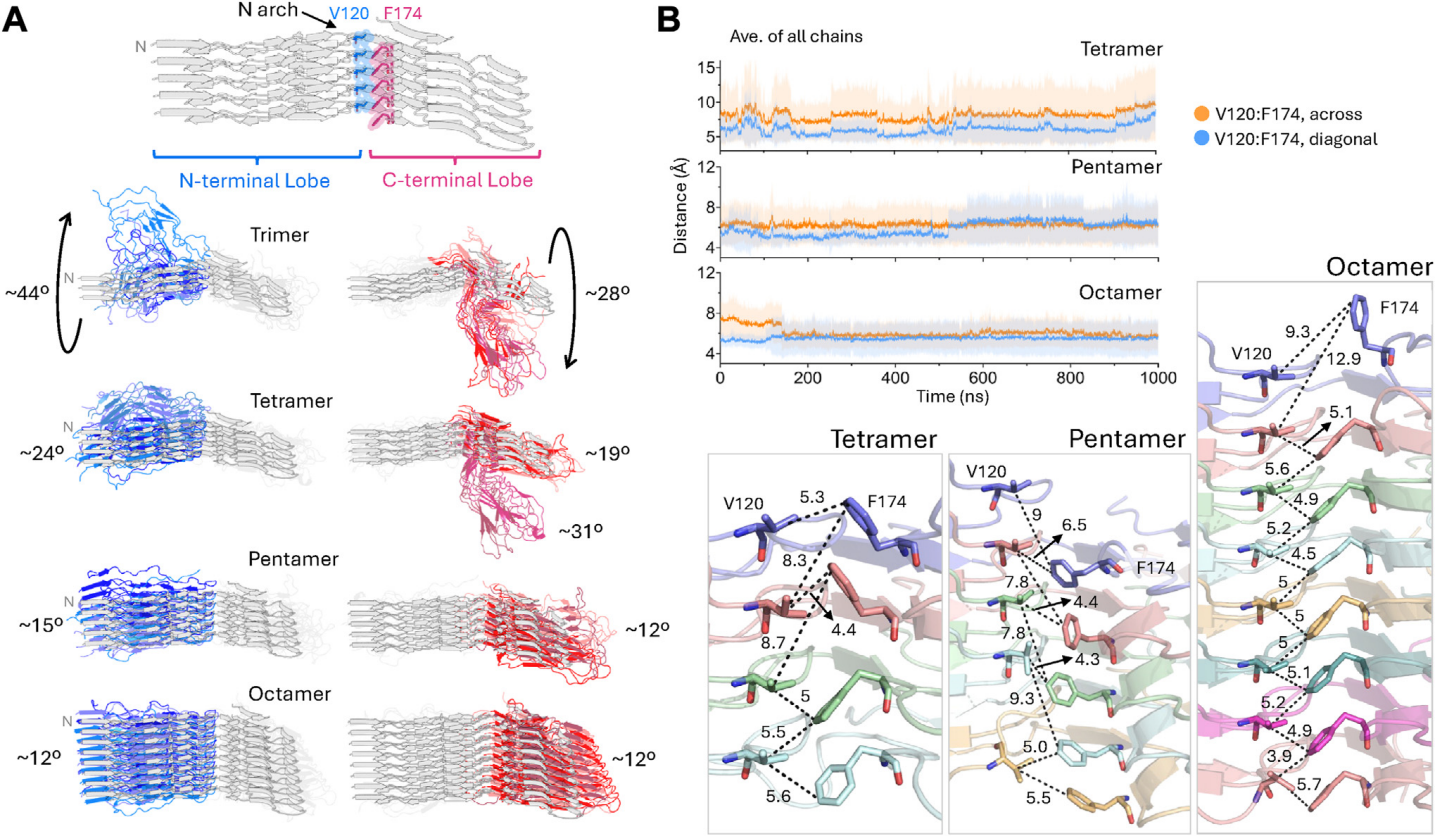
**Domain motions in PrP^Sc^ fragments.***A*, *top* panel identifies the N-lobe:C-lobe interface with residues V120 and F174 shown in licorice representation. Rotation of individual domains and the average degree of rotation for each fragment is shown with each run displayed in shades of *blue* (N-lobe) or *red* (C-lobe). The cryo-EM structure is show in *gray*. *B*, the average distance between the Cβ and Cζ of residues V120 and F174 is plotted as a function of time for the tetramer, pentamer and octamer. The distance across is of V120 and F174 residues on the same chain, and the diagonal distance is of residues F174 on one chain and V120 on the chain below it. In the *bottom* panels, the interface between the lobes for the tetramer, pentamer and octamer of run 1 (last frame) is depicted.

In addition to local fluctuations, PrP^Sc^ oligomers under eight chains in length underwent large domain motions. In the cryo-EM structure, the N- and C-lobes form a staggered interface with a long-range hydrophobic interaction between residues V120 and F174. This allowed for a protrusion of the N-arch on one end of the fibril (Fig. 4*A*; arrows). The distance between the Cβ and Cζ of two residues (V120 and F174, respectively) within the same monomeric chain is 6.1 Å (Fig. 4*B*, across), and the distance between V120 of one chain and F174 in the preceding chain is 5.3 Å (Fig. 4*B*, diagonal). In dimers and trimers (Fig. S8) the distances between the two residues varied between 5.2 to 27 Å, consistent with large conformational heterogeneity and domain instability. The tetramer (Fig. 4*B*) maintained the alignment of the valines at position 120 and the χ_2_ angles of F174 fluctuated between −90° and 90° (Fig. S9*A*). Similarly, a marginal increase in stability was observed in the pentamer as π-π interactions of residue 174 between the chains and with the neighboring histidine at position 176 (Fig. S12) reduced the motion in the V-shaped cleft between the two lobes and decreased the average distance of the V120:F174 interface (Figs. 4*B* and S8*B*). Unlike the tetramer, the pentamer maintained the stagger of the starting structure. The V120:F174 interface is even tighter in the octamer and although the initial ∼170 ns reproduce the values of the starting structure, it is likely that continued twisting of the lobes produced shorter distances in the interface. Interestingly, only some of the MD runs of the fragments (tetramer, pentamer and octamer) recapitulated the signature protrusion of the N-arch seen in the cryo-EM structure; the larger species (decamer and tetradecamer) each maintained the ∼37.9° stagger in both runs (Fig. S10).

Instability in the V120:F174 interface was also apparent by the twisting observed in the N- and C-lobes. The trimer experienced an average of 44 ° (N-lobe) and 28 ° (C-lobe) twisting in the y and z directions, relative to the starting conformation (Fig. 4*A*). Distortion of the largely planar fibrillar surface occurred within the first 250 ns of the simulations and was maintained throughout the duration of the run (Fig. S11). Twisting of the domains was counterclockwise in the N-lobe and clockwise in the C-lobe. In the tetramer, the twist of the two lobes was reduced to ∼24 ° in the N-lobe. However, two populations of the C-lobe twist were identified at either an average rotation of 19 ° or 31 °. This suggests that the tetramer was likely in a metastable state between the two conformational groups. Structural reinforcement provided by hydrogen bonds of an additional chain decreased the twisting in the N-lobe of the pentamer to an average of 12 ° (N-lobe) and 15 ° (C-lobe). In the octamer, the twists in the N- and C-lobes were 12 °, which was sufficient to perturb the interface stagger.

#### Local environment of PrP^Sc^ fragments

Due to large domain motions observed in the dynamics of PrP^Sc^ fragments, a superposition-free metric of the local atomic environment was useful in understanding intra- and inter-molecular atomic distances. We employed the local distance difference test (lDDT) (37), which calculates the fraction of preserved distances (between 0 and 1) of all atoms within the neighborhood of a given residue. The atomic environment is considered preserved in the selected conformation if (within a certain tolerance threshold, see Experimental procedures) it is the same as the corresponding distance in the starting conformation⁠. An LDDT score of 0 indicates that none of the atomic distances were preserved at any threshold. In contrast, a score of one indicates that all the distances were preserved across all the thresholds.

**Figure 5:**
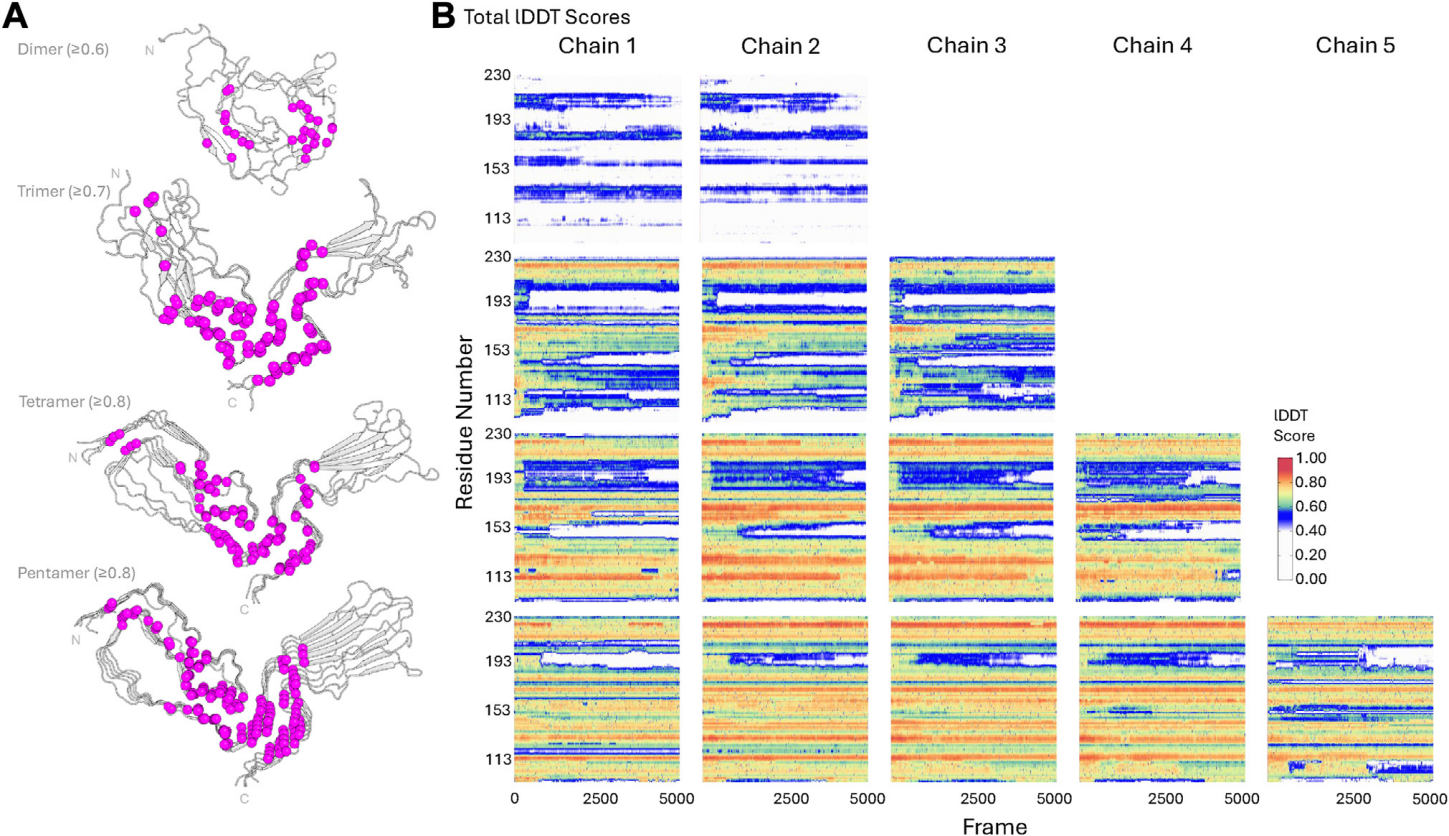
**Conservedinter-atomic distances of PrP^Sc^ fragments.***A*, the lDDT scores of the three or four individual MD runs which were equal to or surpassed the lDDT indicated scores for at least 500 ns (dimer, 0.6; trimer, 0.7; tetramer and pentamer, 0.8) plotted on the backbone structure of the last frame of run 1. The spheres depict all conserved atomic distances (both intra- and intermolecular; *magenta*) *B*, residue-specific lDDT scores of a single run plotted as a function of time. The individual lDDT scores of the other runs are plotted in Figs. S13–S19.

In the dimer, most atomic distances with an average score of ≤0.6 were largely confined around the disulfide bond (C178:C213), as well as a few contacts immediately after the N-arch (Fig. S13). The conformational heterogeneity and varying degrees of disorder amongst dimers in the different MD runs were clearly visible in the intramolecular lDDT plots (Fig. S13*B*). In contrast, the trimer exhibited localized contacts in both the N-lobe and C-lobe (Figs. 5 and S14). Conserved intramolecular distances were observed around hydrophobic residues 121-132, the end of the middle arch up to the disulfide bond, and in the far C-terminus (Fig. 5*A*). The preservation of the atomic environment at the end of the middle-arch (160–164) was likely due to the π-π stacking of TYR161 and TYR162, which created stability between the chains. Another crucial π-π interaction is formed between TYR168 at the latter portion of the N-lobe and TYR225 on the C-terminal tail. The additional chain in the tetramer allowed for further stabilization of the N-arch and the interface between the two ends of the C-lobe, with the π-π interaction serving as a conformational lynchpin (Figs. 5, S12, S15 and S16). The aromatic interaction between TYR168 and TYR225 is ubiquitous among the fragments (≥3 chains). In the pentamer (Figs 5, S17 and S18) and the octamer (Figs. 5 and S19), conserved atomic distances can be seen in the N-arch and the top of the middle-arch, as well as in the C-terminal residues and the residues linking the middle and disulfide arches.

A commonality among all the oligomers is the lack of conserved atomic distances at the tip of the disulfide β-arch, which is mostly composed of threonine residues and two solvent-exposed charged residues K193 and E195 (Fig. 5). There is an internal salt bridge formed by GLU199 and ASP201, however it did not provide enough conformational rigidity to counter the observed fluctuations.

#### Heat stability of a PrP^Sc^ octamer

To further challenge the conformational stability of the prion core architecture, we subjected the octamer to MD simulations at elevated temperatures. MD trajectories of 1 to 2 μs were performed at 300 K (27 °C), 350 K (77 °C), and 400 K (127 °C) with three independent runs at each temperature. The higher temperatures were included in part to test for any tendency of octamers to fragment into smaller subunits.

**Figure 6:**
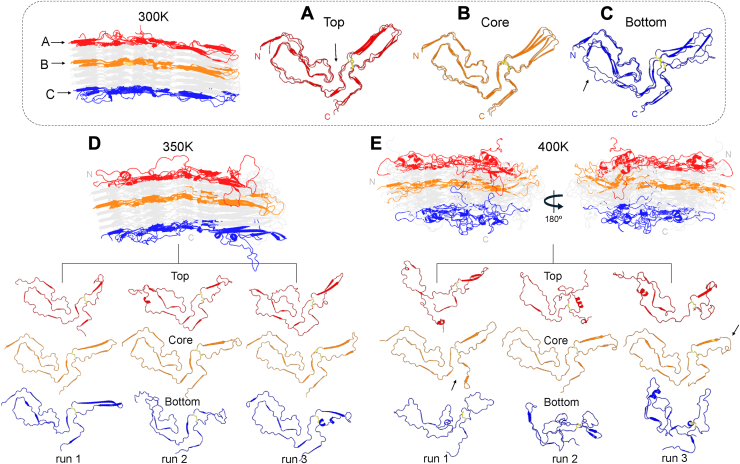
**Heating of octameric PrP^Sc^.** The last frame from simulations of the octamer at 300 K, where the (*A*) *top*, (*B*) core and (*C*) *bottom* chains of each run are overlayed. The last frame from simulations of the octamer at 350 K (*D*) and 400 K (*E*) showing the *top*, core and *bottom* chains of each run.

As seen in Figure 6, the PrP^Sc^ octamers maintained their overall PIRIBS architectures even at the higher temperatures. At 300 K, the octamer experienced minor structural perturbations with most of the variability being confined to the terminal chains (Fig. 6, *A* and *C*, and S20). Despite flexibility in the top chain, the conformation of the N-arch was preserved from residue 93 to the start of the middle arch at residue 140 (Fig. 6*A*). The structural plasticity observed in the middle arch was accompanied by a loss of secondary structure, which is also seen in the tip of the disulfide β-arch. The disulfide bridge at the beginning of the disulfide β-arch served to locally stabilize the residues participating in the hydrophobic interface between the N- and C-lobes of all the chains (Fig. 6*A*, arrow). Conformational reorganization also occurred in the bottom chain; however, unlike the top chain most of the loss in β-sheet character was in the N-lobe, specifically in the central lysine cluster (CLC; residues 100, 103,105,109) and the middle arch (Fig. 6*C*, arrow). As seen in Figure 6*B*, the core of the octamer displayed all the structural elements of the starting conformation with minimal changes.

At 300 K, thermal motion allowed for the V-shaped cleft between the N- and C-lobes to widen from 71 Å in the cryo-EM structure to an average of 77 Å observed during the MD simulations (Fig. S21). After reaching a cleft width of 82 Å, the two lobes oscillated in relation to each other by (±) 1 to 3 Å for the duration of the simulations. These trends were observed in all three trajectories as illustrated by the overlayed structures in Figure 6, *A–C*, and the backbone RMSD plots in Fig. S20.

At 350 K, most secondary structural elements throughout the internal chains of the octamer remained intact with little deviation in the N-terminal motifs. In one of the runs, twisting at the end of the disulfide β-arch was visible, which caused a considerable increase in the angle between the N-and C-lobes (Figs. 6*D*, core and S21). Conformational stability in the core is likely reinforced by intermolecular hydrogen bonding. The top chains exhibited prominent reorganization of the middle arch in which one side of the arch folded over the other. Portions of the original conformation of the head of the N-arch remained discernible in two of the runs, but there were major reductions in β-sheet character throughout the top chains. In one of the bottom chains, there was partial unfolding in the far N-terminus, specifically in the steric zipper and CLC regions, as well as in the disulfide β-arch. Unfolding of regions of the disulfide β-arch enabled the formation of transient α-helices. The higher temperature produced substantial variability between runs, as illustrated by average backbone RMSD values at 16 Å (Fig. S20). As in the 300 K simulations, the cleft formed by the N- and C-lobes of the octamer at 350 K widened in comparison to the cryo-EM structure. The average width is centered at ∼88 Å with a ± 2 Å fluctuation (Fig. S21).

At 400 K, the aRML PrP^Sc^ octamer experienced greater unfolding of both N- and C-lobes. The almost fully disordered top chain made various short-lived inter- and intra-molecular interactions throughout the >2 μs simulations. Interestingly, the core of the octamer maintained an ordered N-lobe (Fig. 6*E*, core). The C-lobe of the core was variable throughout the three runs; however, constant in all the core chains was a widening of the cleft between the N- and C-lobes and twisting of the tip of the disulfide arch. In the first run, one of the core chains exhibited separation of the far C-terminus from the C-lobe. Like the top chain, the bottom chain underwent global unfolding. The bottom chain exhibited the most disorder with hardly any discernible structural motifs (Fig. 6*E*, bottom). In both the second and third runs, the disordered N-terminal tail engaged in nonspecific interactions with neighboring chains, including the top chain of the octamer by way of the N- and C-lobe cleft. Despite regions of disorder in octamers of both the 350 K and 400 K runs, especially in the terminal monomers, none of the chains were seen dissociating from the PrP^Sc^ octamers in this time frame and in the presence of periodic boundary conditions.

#### Heat stability of PrP^Sc^ 25-mers from three prion strains

**Figure 7:**
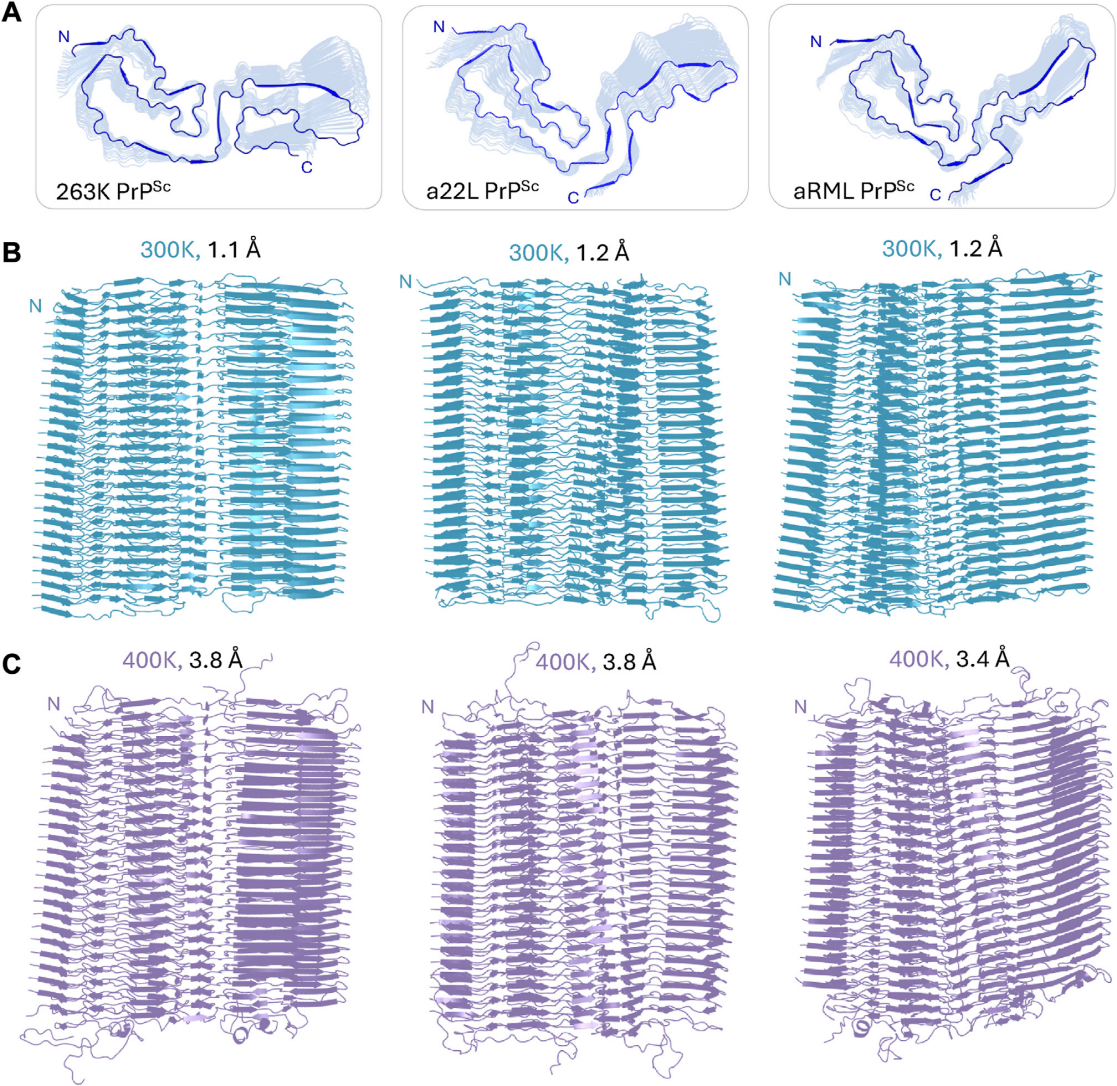
**Structures of 25-mers of three PrP^Sc^ strains.***A*, *top* view of three PrP^Sc^ fibril strains 263K, a22L, aRML of the last frame from a single simulation run. *B*, side view of PrP^Sc^ fibrils at 300 K (263K; 260 ns, a22L; 260 ns, aRML; 310 ns) of the last frame from a single simulation run with respective average backbone RMSD. *C*, side view of PrP^Sc^ fibrils at 400 K (263K; 470 ns, a22L; 480 ns, aRML; 580 ns) of the last frame from a single simulation run with respective average backbone RMSD.

Trends observed in the heating simulations of the aRML PrP^Sc^ octamers were also recapitulated in larger species (25-mers) (Figs. 7 and S22). Fragments composed of 25 monomers in length of aRML PrP^Sc^ and two additional strains (263K PrP^Sc^; hamster and a22L PrP^Sc^; mouse) were simulated at 300 K and 400 K. At 300 K, fibrils maintained all structural motifs, including the interface stagger and did not exhibit domain twisting. The PIRIBS architecture was retained, and no breaks were visible in the core of the fibrillar structures. At 400 K however, the terminal chains were largely unfolded and readily adopted transient helices usually in segments that are helical in PrP^C^. In the three runs performed for each strain at 400 K, the bottom chains were more mobile than the top, frequently folding into α-helices, however more simulations are necessary to establish specific differences between the top and bottom chains. As in the 300 K simulations, the cores of the fibrils did not experience breaks and maintained all initial structural motifs under these conditions.

## Discussion

### Minimum stable oligomeric unit of a prion fibril core conformation

Deciphering the factors that control the sizes of pathological protein aggregates is fundamental to the understanding of mechanisms by which such abnormal assemblies form, propagate, and cause disease. Even femtogram quantities of PrP prions inoculated into a highly localized site can, and must, spread over time to cause clinical disease. Accumulating evidence suggests that smaller proteopathic aggregates may be more infectious and toxic than much larger amyloid plaques, which may be relatively inert “dumping grounds” for millions of indigestible amyloid fibrils. Although it is often suspected or assumed that small disease-associated oligomers have different core conformations than amyloid fibrils, our current computational analyses suggest that tetrameric units of a known infectious prion structure can maintain most of the core conformational features of much larger prion fibrils, including motifs of the templating surface that is responsible for strain-dependent prion fibril elongation. The tetramer and larger oligomers likely gain progressively greater stability through cumulative intermolecular hydrogen bonding, which reinforces important structural motifs (N- and middle arches), further enabling the formation of long-range interactions.

### Prion infectivity and the cores of PrP fibrils

The cryo-EM structures of highly infectious *ex vivo* mammalian prions have ordered fibril cores that extend from residues 93-95 to 226-230, *i.e.*, spanning the major N- and C-lobes of each monomeric unit (10, 11, 12, 15, 16, 17). However, fibrils from human patients with Gerstmann-Straussler-Scheinker syndrome caused by the *PRNP* mutation encoding a phenylalanine to serine (F198S) substitution in PrP have much smaller ordered cores spanning residues 80-141 (38). Brain extracts from these patients have been shown to be transmissible to bank voles (39) but not to non-human primates or transgenic mice overexpressing human PrP^C^ (40). The structures of synthetic recombinant PrP amyloid fibrils have also been determined (41, 42, 43) but these fibrils are not known to be infectious. These synthetic fibrils have much smaller ordered cores within residues within either the N- or C-lobes of *ex vivo* prions, but not both. Many other synthetic PrP fibrils with relatively small protease-resistant cores (of undetermined structure) have been shown to be at least several orders of magnitude less infectious than *bona fide* tissue-derived prions (*e.g.* (44, 45, 46, 47)). These findings, along with the demonstrated infectivity of synthetic fibrils of a C-terminally truncated mouse PrP construct of residues 23-144 (48), indicate that fibril assembly *via* residues from within those spanned by the N-lobe of the aRML core can sometimes be infectious. In contrast, to our knowledge, fibrils with cores corresponding only to the C-lobe of rodent prion fibrils have not been shown to be transmissible. In this context, we found that the N-lobe of the aRML core structure is more stable than the C-lobe in our simulations. Greater flexibility in portions of the C-lobe is consistent with the lower resolution seen towards the tips of the disulfide arches in all infectious *ex vivo* prion structures to date (10, 11, 12, 13, 14, 15, 16, 17). Conversely, the disulfide bond at the base of the disulfide arch, which is maintained during prion conversion contributes significantly to the structural stability of the region. Even in the smaller, less structured fragments (*e.g.* trimers), the disulfide bond maintains its rigidity and that of nearby residues. Although we initially suspected that conformational plasticity in the disulfide arch might be due to the N-linked glycans, the fact that we have seen reduced stability in this arch even in the absence of such glycans *in silico* suggests that this domain is inherently less stable than more N-proximal domains, at least in the context of the RML structure. Interestingly, two unglycosylated recombinant human PrP fibrils, one with the wildtype PrP sequence and the other with the familial prion disease-linked E196K PrP mutation have fibrillar conformations with little evidence of plasticity at the tips of the disulfide arches (42, 43). However, these fibrils are composed of two protofilaments that contact one another *via* the disulfide arch tips in a way that would be precluded by the presence of glycans at Asn197. Also, since these fibrils were formed spontaneously without any constraints imposed by glycans, they might be able to adopt disulfide arch conformations and 2-protofilament fibrils that are more conformationally stable than those in *ex vivo* prions, but incompatible with the incorporation of glycosylated PrP^C^ molecules as would be required for propagation upon inoculation into wildtype hosts.

Consistent with the latter notion are observations that when certain synthetic PrP fibrils are inoculated into transgenic mice expressing similar amounts of both wild-type and underglycosylated GPI-anchorless PrP^C^, it is mainly the latter that is converted to protease-resistant forms *in vivo* (without causing overt clinical disease) (49). Thus, it is conceivable that when prions are injected into wild-type hosts the most efficient interactions between their templating surfaces and PrP^C^ occur *via* N-lobe residues rather than the glycosylated, GPI-anchored C-lobes, where steric clashes between bulky post-translational modifications may complicate initial contacts.

### Implications for mechanism of PrP conversion and prion elongation

The greater fluctuations of the terminal chains of prion oligomers of all sizes (Figs. 3, 6 and 7) may have implications for the mechanism by which new monomers are added onto growing fibrils. Whether the prion templates interact directly with native PrP^C^ molecules, partially unfolded intermediates, or oligomers thereof, is unclear. However, mobility in the templating surfaces may enhance options for interactions between PrP^Sc^ and incoming PrP molecules, potentially reducing entropic costs and activation energy barriers in the conversion process. Also, plasticity in the terminal monomers might facilitate conformational shifts in the template to generate alternative prion variants or strains. The fact that our MD simulations revealed no tendency of the aRML fibril core structures to disassemble into dimeric to tetrameric “elemental bricks”, even at elevated temperatures, appears to be inconsistent with previous reports of the behavior of wildtype RML and other prion strains in brain homogenates under certain detergent or chaotropic conditions (31, 32). It remains possible that with increased sampling time, disassociation of terminal monomers, dimers, or other subunits might be observed under our saline, neutral pH conditions, but our current data suggest that detached dimers (or, by inference, monomers) would retain little of the original aRML PrP^Sc^ conformation. The basis for the apparent inconsistencies of our results and the previous reports of prion disassembly (31, 32) is unclear but might relate to the following issues: First, our MD simulations were performed on the “naked” fibril core structures without detergents or chaotropes and in the absence of N-terminal domains, glycans, and GPI anchors that are present on wildtype RML prions. Thus, it remains possible that these elements of wildtype RML prions could potentiate fragmentation in the presence of detergents or urea, for example by imposing periodic stresses that destabilize the PIRIBS core structure. We note, however, that we and others have provided evidence that triantennary N-linked glycans can be accommodated on each chain of a PrP PIRIBS fibril core structure without apparent destabilization (50, 51). Secondly, the disassembly of RML prions observed in brain extracts might be facilitated by hydrolases, chaperones, disaggregases and lipids. In the brain itself, physiological activities that might assist fragmentation include proteostatic systems; innate immune factors; acidification of autophagic vacuoles, endosomes, and lysosomes; interactions with cytoskeletal elements and motors; and membrane dynamics as applied to wild-type GPI-anchored fibrils (10). Third, as shown in our companion paper (33), experimental artifacts might provide alternative explanations for data previously interpreted as evidence of spontaneous PrP^Sc^ disassembly into dimers-tetramer (30, 31, 32). The latter impressions were based primarily from size exclusion chromatography, the interpretation of which can be confounded by particle binding to column matrices, making particles appear much smaller than their actual size.

### Comparing the *in vitro versus in silico* thermal stabilities of RML prions

Although the internal chains of the octameric aRML model maintained many conformational features at 400 K (126 °C) in our simulations, their disulfide arches, as well as the entire sequence of the terminal chains, deviated markedly from their starting conformations in each of three independent runs. Interestingly, a previous study showed that wild-type RML prions in brain homogenates are highly sensitive to 2 h of exposure to 98 °C in terms of both infectivity and protease-resistance (52). Taken at face value, the comparison of these *in silico* and *ex vivo* studies suggests that heat-induced distortions of the disulfide arches and terminal chains of a prion particle may be sufficient to corrupt the terminal templating surfaces, and hence the infectious self-propagating activity of aRML prions. Alternatively, or additionally, the presence of N-terminal domains, post-translational modifications, or other brain components might contribute to the thermolability of aRML prions in crude brain homogenates.

### Conclusion

In attempting to understand prion pathogenesis and to envision rational approaches to prion therapeutics and vaccine development, it is important to establish the range of prion particle sizes as well as their relative stabilities and conformational dynamics. Because detailed empirical analyses of small prion oligomers are exceptionally difficult due to their size heterogeneity and relative scarcity in most tissue-derived prion preparations that retain infectivity, we have opted to obtain initial insights from computational approaches based on the high-resolution structure of a brain-derived prion fibril. Overall, our results provide evidence that the PIRIBS-based prion fibril core structure has considerable stability even in oligomeric units as small as tetramers, and to a far lesser extent, trimers. However, larger oligomers showed no signs of spontaneous disassembly into smaller units, at least *in silico* without unstructured N-terminal PrP domains, posttranslational modifications, or non-PrP ligands. This result is consistent with our failure to detect spontaneous disassembly of PrP^Sc^ in brain homogenates into small oligomers in detergents in our companion paper (33), as well as in many prior studies described therein.

## Experimental procedures

### System setup

The initial structure of the aRML prion fibril was obtained from the RCSB Protein Data Bank (PDB ID: 7TD6) (11). Multiple copies of aRML were opened in ChimeraX 1.9 (53) and aligned to the cryo-EM map obtained from Electron Microscopy Data Bank (54) (EMD-25824) using the fit in map functionality. Fragment sizes were modified accordingly.

### All-atom molecular dynamics simulations

After each aRML fragment was created, GLYP and CNEU patches were added to each of the monomer termini using CHARMM-GUI (55) and the protonation states of all titratable residues were set as ASP, GLU, HIS: deprotonated; ARG, LYS: protonated. The termini of the 25-mers were patched with ACE and CNEU and all titratable residues were (de)protonated according to a system pH of 7.0. The DISU patch was added to create the disulfide bond between residues 178 and 213 of each chain. All systems were then solvated with TIP3P water in an orthorhombic water box (the sizes of which are listed in Tables S1 and S2) and 150 mM NaCl was added with neutralizing counterions. Initial scripts were generated with CHARMM-GUI and all simulations were performed with NAMD 3.0-beta6-CUDA (56) and the CHARMM 36 m (57) protein force field with WYF-cation correction (58). Each system was energy-minimized with a gradient of backbone and sidechain restraints ranging from 10 kcal/mol/Å to 0 kcal/mol/Å in iterative runs of conjugate gradient minimization for a total of 85,000 steps. A 3 ns NVT equilibration simulation with backbone restraints was performed for each fragment at a temperature of 300 K maintained with a Langevin thermostat. The simulations advanced at a timestep of 1 fs and the particle mesh Ewald algorithm was used to calculate long-range electrostatics. Non-bonded interactions had a cutoff of 12 Å and the rigid bond algorithm was applied to all bonds containing hydrogen atoms. Subsequently, 3 ns NPT equilibration runs were performed with backbone restraints where the pressure was kept constant with the Langevin piston method. This was then followed by a 5 ns NPT equilibration run without restraints. The NPT production simulations advanced at a 2 fs timestep for at least a duration of 1 μs and until simulations converged *via* backbone RMSD. All simulations were performed at least twice. For the heating simulations at 350 K and 400 K, an additional heating step was introduced before the equilibration runs where the temperature increased by 1 K every five steps.

### Structural analysis

All simulations were visualized with the VMD 1.9.3 (59) and Pymol 2.6.0 (http://www.pymol.org/pymol) visualization packages. Root mean square deviations and fluctuations were computed with the Bio3D protein analysis package (60) and VMD. Secondary structure assignment was performed with the CHARMM/c46b1 molecular dynamics platform (61) and the coor sec functionality and the dss function in Pymol. Radius of gyration and solvent accessible surface area (probe radius 1.4 Å) were computed with the correl functionality. CHARMM was also utilized to compute the degree of twist in the N-and C-lobes with the coor orie functionality. The angle of rotation was determined by first aligning the initial fibril structure horizontally along the x-axis and rotating the center of mass of either the N- or C-lobes of a subsequent frame to the x-axis.

### Local distance difference test (lDDT) analysis

lDDT analysis was performed with OpenStructure (37, 62). The cryo-EM structure was used as the reference structure and the lDDT scores of each residue at every frame were computed and plotted as a function of time. The final lDDT score is the average of four fractions computed using the thresholds 0.5 Å, 1 Å, 2 Å, and 4 Å. An lDDT score of 0 indicates that none of the atomic distances were preserved at any threshold. In contrast, a score of one indicates that all the distances were preserved across all the thresholds. For a given lDDT cutoff (0.6; dimer, 0.7; trimer, 0.8; tetramer-octamer), the fraction of preserved distances was calculated and mapped onto the protein backbone. Residues above a certain cutoff, for least 2500 frames (500ns) or higher, in all the copies of a simulation were identified across the entire fibril (total).

## Data availability

All data are included in the manuscript and/or supporting information. The input files and trajectories will be available upon reasonable request to the authors.

## Supporting information

This article contains supporting information.

## Conflict of interest

The authors declare that they have no conflicts of interest with the contents of this article.
