# Supplementary material for: How short is too short for amyloid fibrils?: Molecular dynamics of oligomers of infectious prion core structures
Source: J Biol Chem. 2025 Jun 19;301(7):110390. doi: 10.1016/j.jbc.2025.110390 (PMC12301783; doi:10.1016/j.jbc.2025.110390)
Supplement: Supporting information [file mmc1.pdf]

Oligomer size and the conformational integrity of prion amyloid fragments

Efrosini Artikis[1]*, Amitava Roy[2], Byron Caughey[1]*

SUPPORTING INFORMATION

Table S1. Simulation Details of aRML Fibrils

aRML					
	boxsize (Å)	temp (K)	run	frames	time (μs)
2mer	186 x 148 x 148	300	1	6214	1.24
			2	5192	1.04
			3	5212	1.04
			4	7206	1.44
3mer	186 x 152 x 152	300	1	5065	1.01
			2	5069	1.01
			3	5319	1.06
			4	6385	1.28
4mer	186 x 154 x 154	300	1	5443	1.09
			2	7664	1.53
			3	5452	1.09
			4	5228	1.05
5mer	186 x 156 x 156	300	1	5129	1.03
			2	6235	1.25
			3	5920	1.18
8mer	210 x 160 x 160	300	1	5000	1.00
			2	5000	1.00
			3	5000	1.00
		350	1	5000	1.00
			2	5000	1.00
			3	5000	1.00
		400	1	11772	2.35
			2	12203	2.44
			3	12191	2.44
10mer	210 x 166 x 166	300	1	5642	1.13
			2	5657	1.13
14mer	210 x 172 x 172	300	1	5086	1.02
			2	5746	1.15
25mer	250 x 230 x 250	300	1	1533	0.31
			2	1394	0.28
		400	1	2572	0.51
			2	2905	0.58
			3	2797	0.56

Table S2. Simulation Details of 263K and a22L Fibrils

a) 263K

	boxsize (Å^3)	temp (K)	run	frames	time (μs)
25mer	250	300	1	1310	0.26
			2	1317	0.26
		400	1	1292	0.26
			2	2341	0.47
			3	1285	0.26

b) a22L

	boxsize (Å^3)	temp (K)	run	frames	time (μs)
25mer	250	300	1	1304	0.26
			2	1314	0.26
		400	1	2377	0.48
			2	2376	0.48
			3	2326	0.47

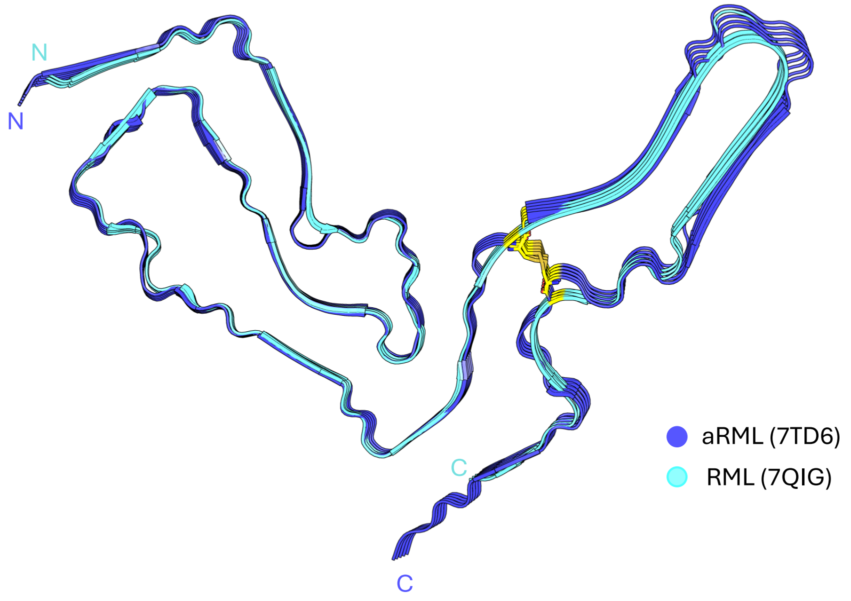

Figure S1. Comparison of cryo-EM-based cross-sections of anchorless RML (aRML; residues 93-230) and wild-type RML (RML; residues 94-225) PrP^{Sc} fibrils. The disulfide bridge between cysteine residues 178 and 213 is in yellow.

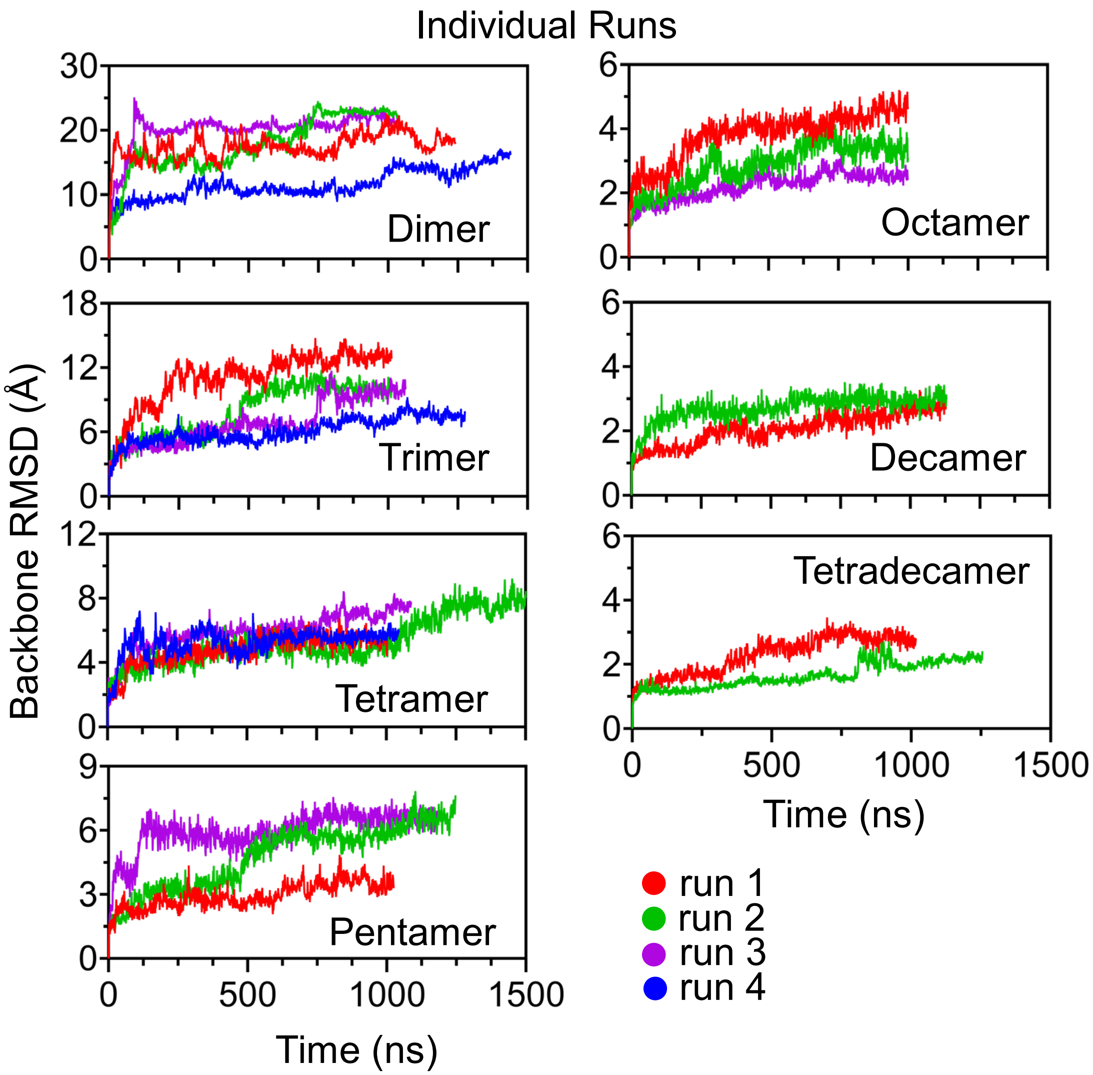

Figure S2. **Backbone RMSD of individual runs**: dimer, 4 runs; trimer, 4 runs; tetramer, 4 runs; pentamer, 3 runs; octamer, 3 runs; decamer, 2 runs; tetradecamer, 2 runs. Backbone RMSD computed in reference to the first frame of each production run. Note the different scales in the RMSD y-axis. The plots of the octamer RMSDs are of the 300K simulations are also depicted in Figure S20B for comparative purposes.

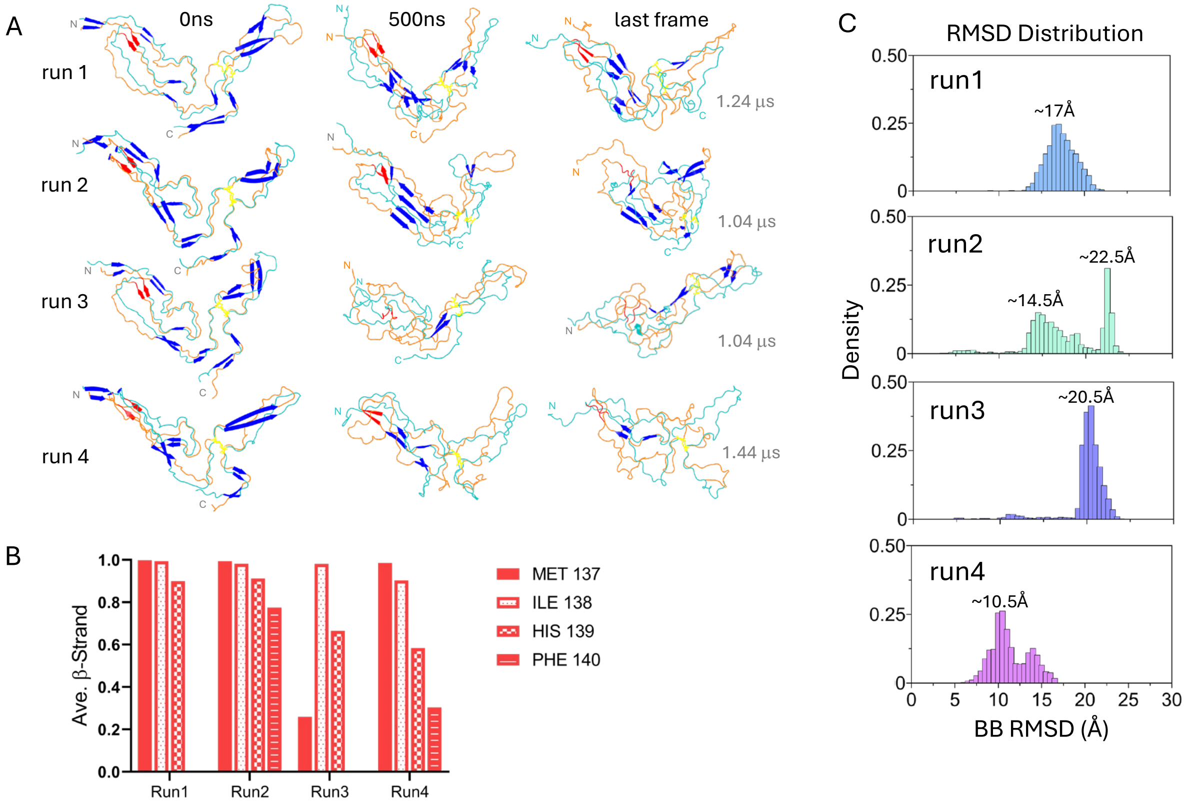

Figure S3. Comparison of 4 individual dimer simulation runs. A) Frames of dimer extracted from each trajectory at 0, 500 and >1000 ns time points. The chains of the dimer are colored in turquoise and orange; β-sheets are highlighted in dark blue; disulfide bridges are indicated in yellow; and the parallel β-strand formed by residues 137-140 is shown in red. The secondary structural assignment was performed in Pymol with the dss command. **B)** The average frequency of β-strand formation in both chains of the dimer is plotted by residue and was assigned with the DSSP algorithm through the coor sec functionality in CHARMM. **C)** Backbone (BB) RMSD distribution for each dimer simulation.

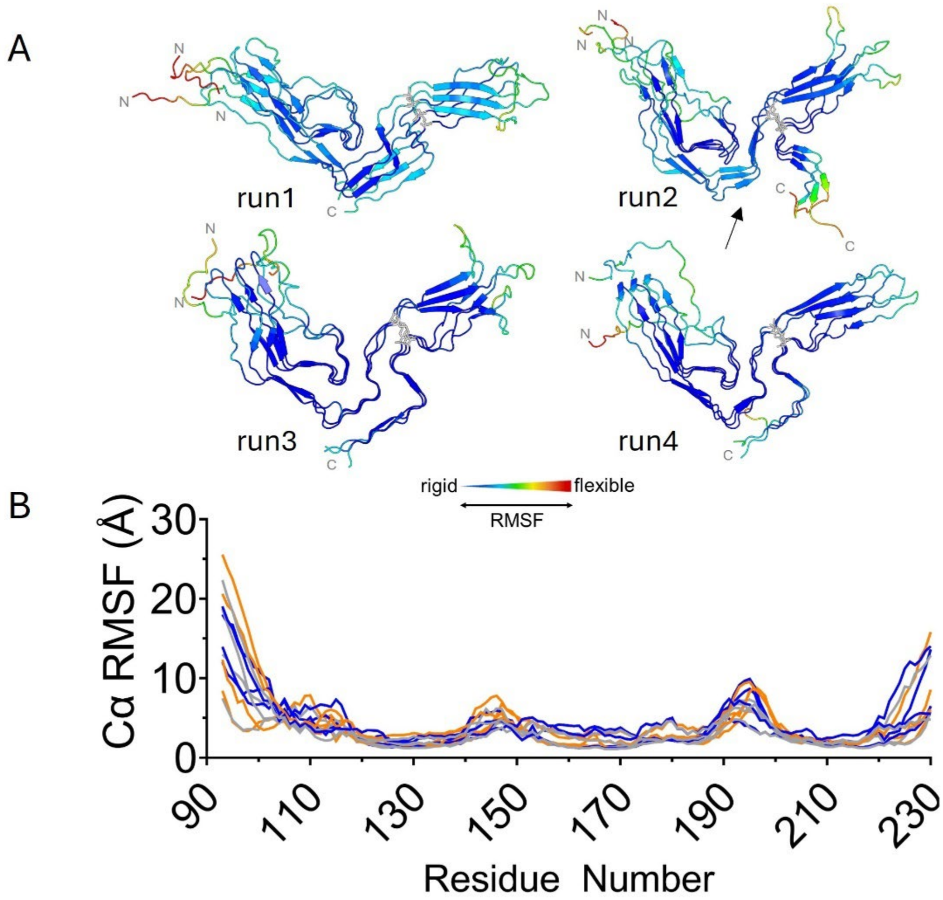

Figure S4. RMSF analyses of trimer. A) Last frames of simulation runs 1-4 with backbone color gradient displaying Cα RMSF values from low (blue, 1.2Å) to high (red, cutoff at 15Å). The disulfide bond (C178:C213) is depicted in gray. **B)** The Cα RMSF values of each residue in each chain from each run are also plotted and the line colors indicate the order of the chains (blue; top chain, gray; middle chain, orange; bottom chain). The arrow marks residues on the end of the middle arch.

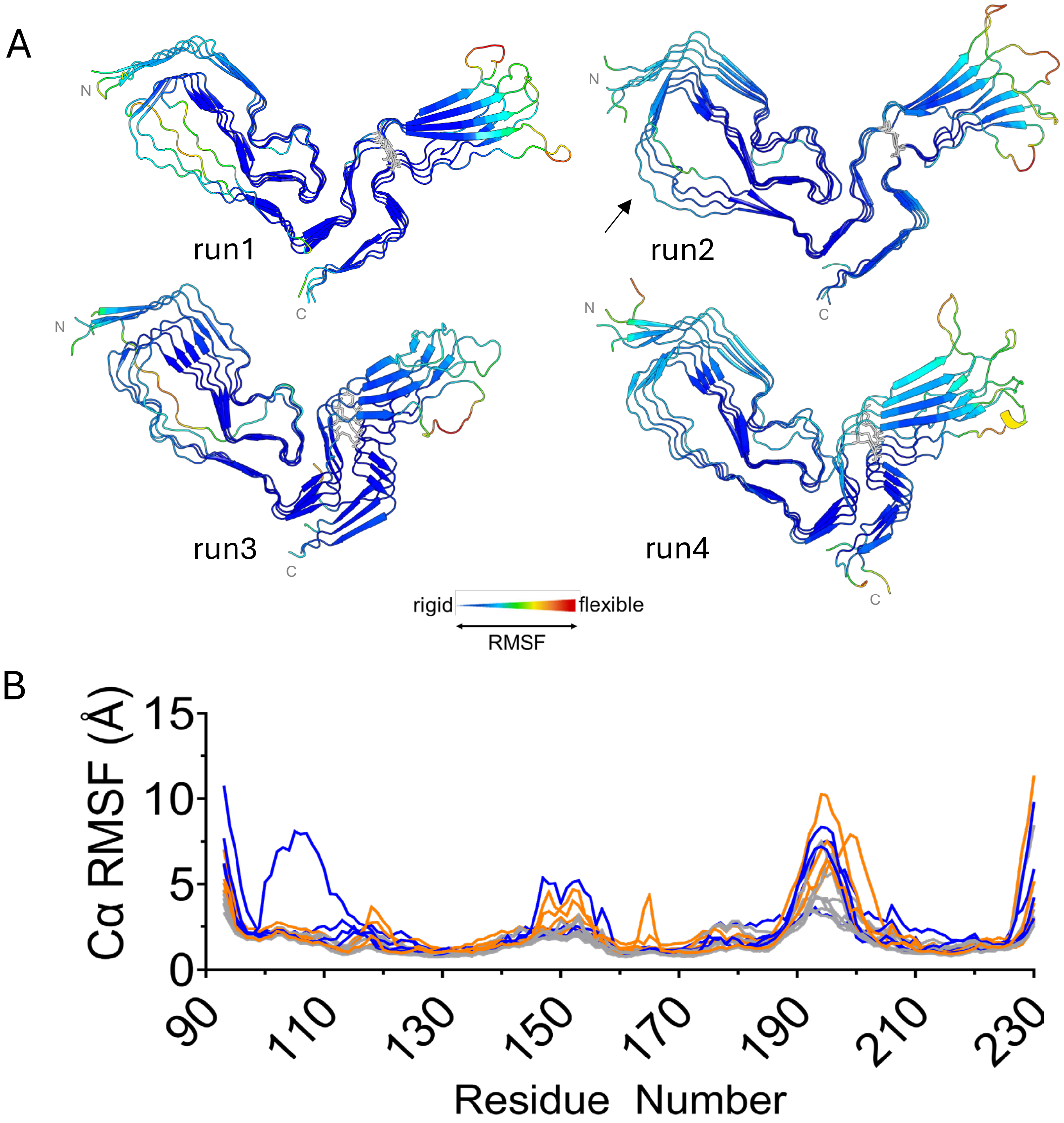

Figure S5. RMSF analyses of tetramer. A) Last frames of 4 tetramer simulations with the backbone color gradient displaying Cα RMSF values from low (blue) to high (red). The disulfide bond (C178:C213) is depicted in gray. **B)** The Cα RMSF values of each residue in each chain from each run are also plotted and the line colors indicate the order of the chains (blue, top chain; gray, core chains; orange, bottom chain). The arrow marks residues on the outward facing flank of the middle arch.

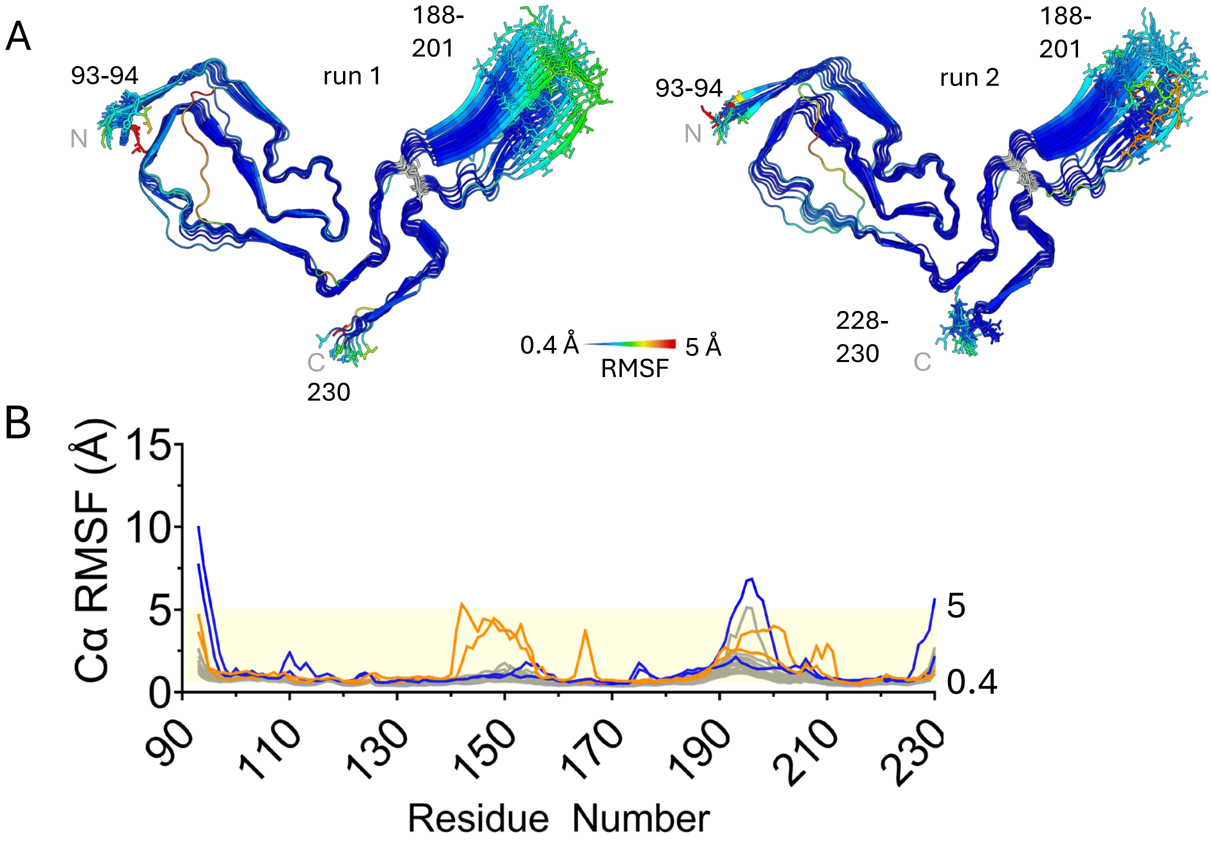

Figure S6. RMSF analyses of decamer. A) Last frames of decamer simulation runs 1 and 2 with the backbone color gradient displaying Cα RMSF values from 0.4 Å (blue, average minimum) to high 5.0 Å (red, average maximum). The disulfide bond (C178:C213) is depicted in gray. **B)** The Cα RMSF values of each residue in each chain from each run are also plotted and the line colors indicate the order of the chains (blue, top chain; gray, core chains; orange, bottom chain). The yellow shading represents the RMSF values within the bounds of 0.4 and 5 Å.

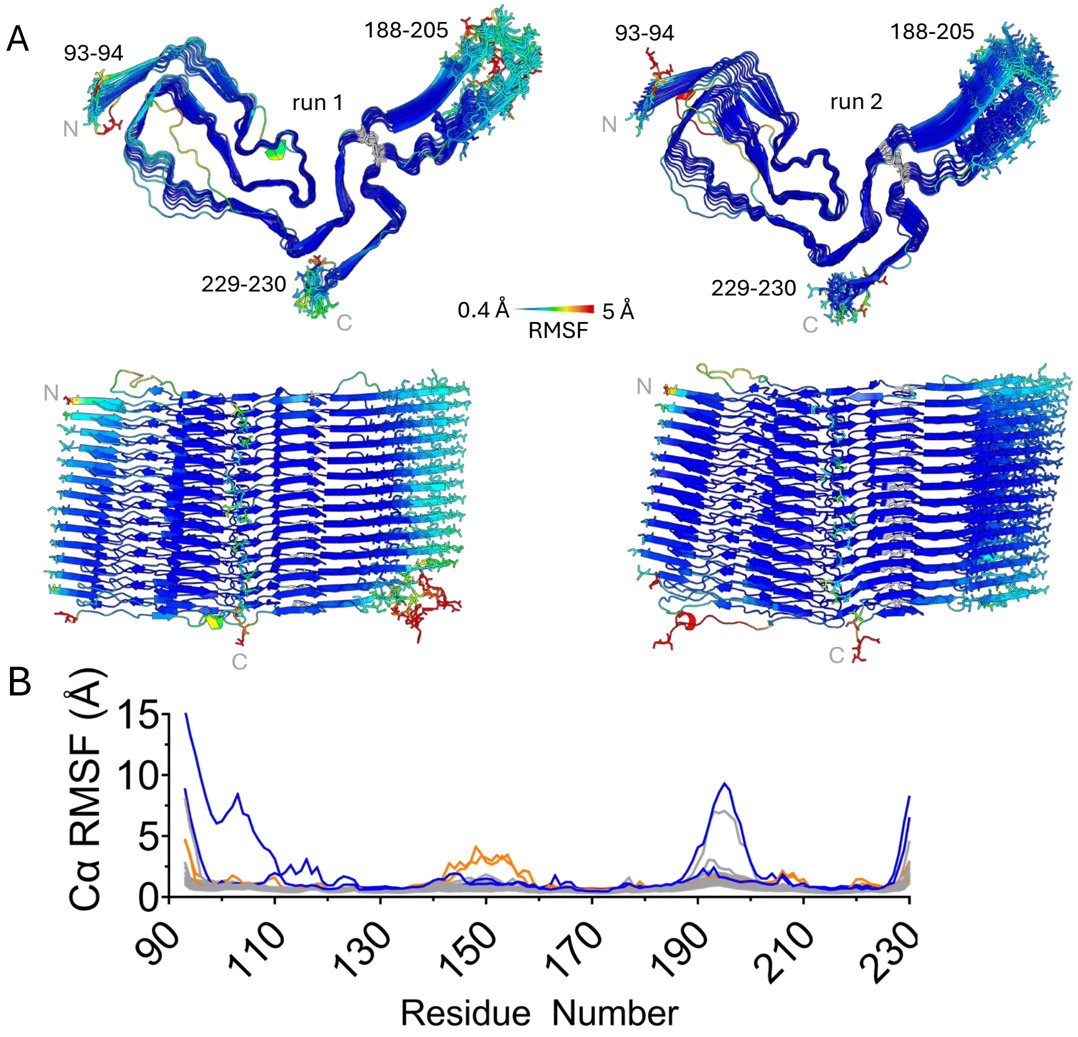

Figure S7. RMSF analyses of tetradecamer. A) The last frames of simulation runs 1 and 2 with the backbone color gradient displaying Cα RMSF values from 0.4 Å (blue, average minimum) to high 5 Å (red, average maximum). Everything larger than the average maximum RMSF of 5 Å is colored red, and anything less than the average minimum of 0.4 Å is colored blue. The disulfide bond (C178:C213) is depicted in gray. **B)** The Cα RMSF values of each residue in each chain from each run are also plotted and the line colors indicate the order of the chains (blue, top chain; gray, core chains; orange, bottom chain).

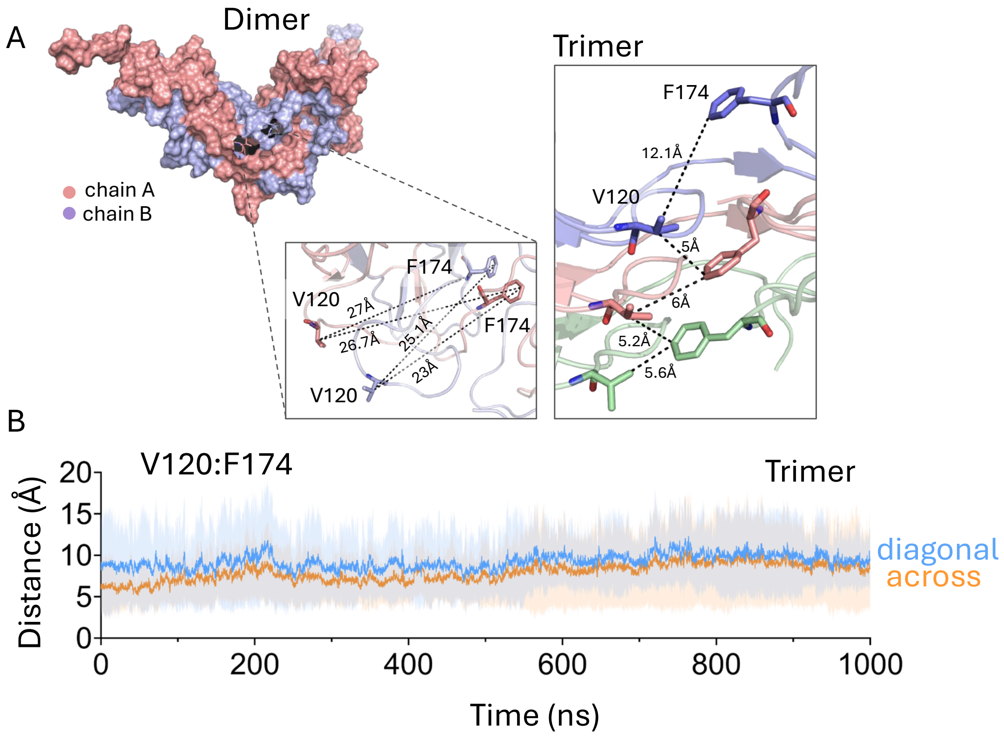

Figure S8. Distance between V120 and F174 in dimer and trimer. **A)** Structure of the dimer (run 1) in surface representation with distances between V120 and F174 (atoms Cβ and Cζ, respectively), depicted in the insets for dimer and trimer . **B)** Average distance between V120 (atom Cβ) and F174 (atom Cζ) of the same chain (across) and distance of F174 (Cζ) of one chain and V120 (Cβ) of the subsequent chain (diagonal) plotted as a function of time for the last 1μs of 4 simulations.

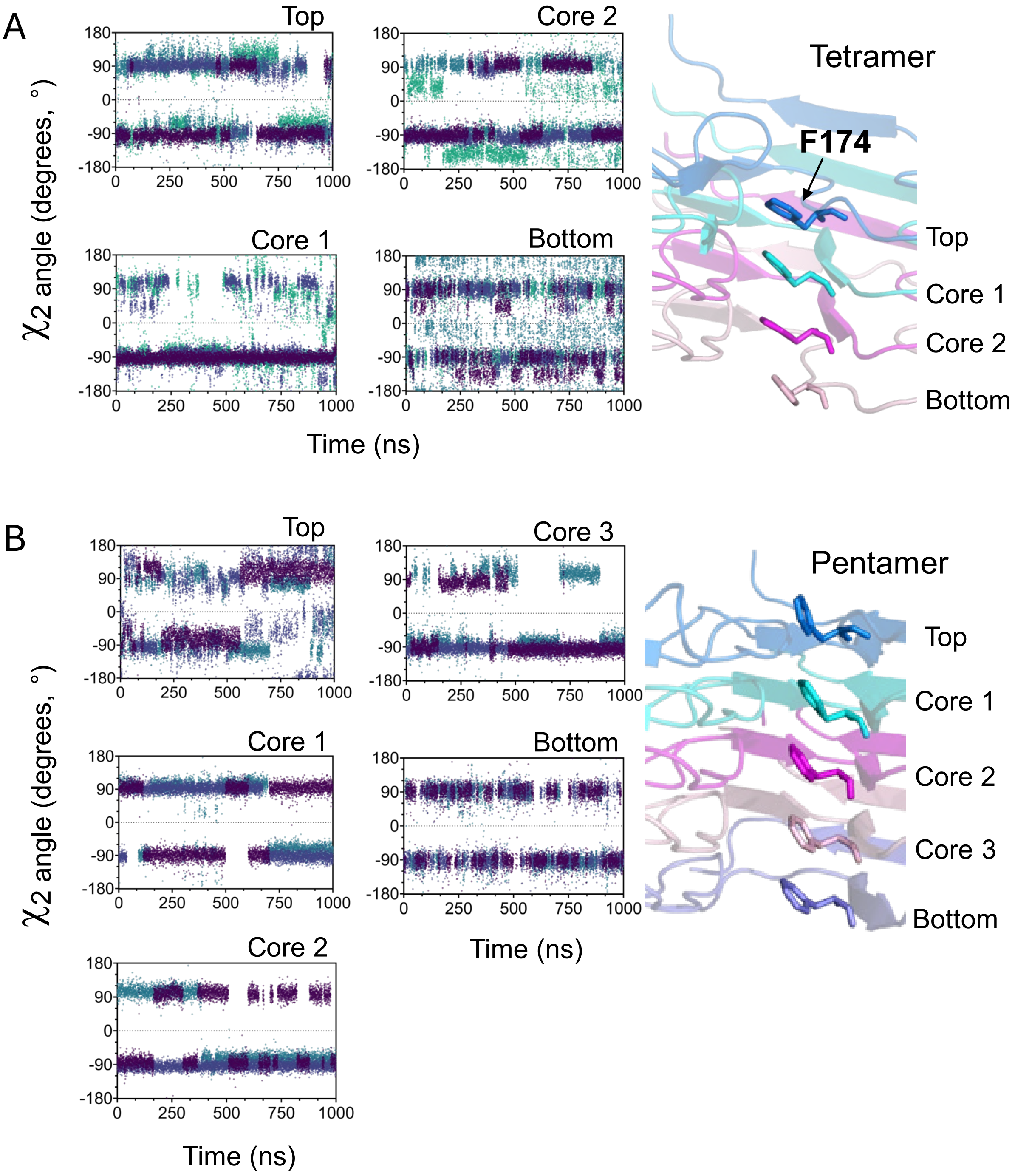

Figure S9. Comparison of χ2 angle of F174 in tetramer vs pentamer. The χ_2 angle of F174 is plotted with respect to time (ns) and each color represents the last 1μs of a single MD run (run 1, green; run 2, blue; run 3, turquoise; run 4 purple). **A)** The F174 χ_2 angle is plotted for either the top, core, or bottom chains of the last 1 μs for each of the 4 tetramer runs. A schematic on the right illustrates the chain designations. **B)** Three runs of the pentamer are plotted in each panel, depicting the F174 χ_2 angle in either the top, core, or bottom chains. A schematic on the right illustrates the chain designations.

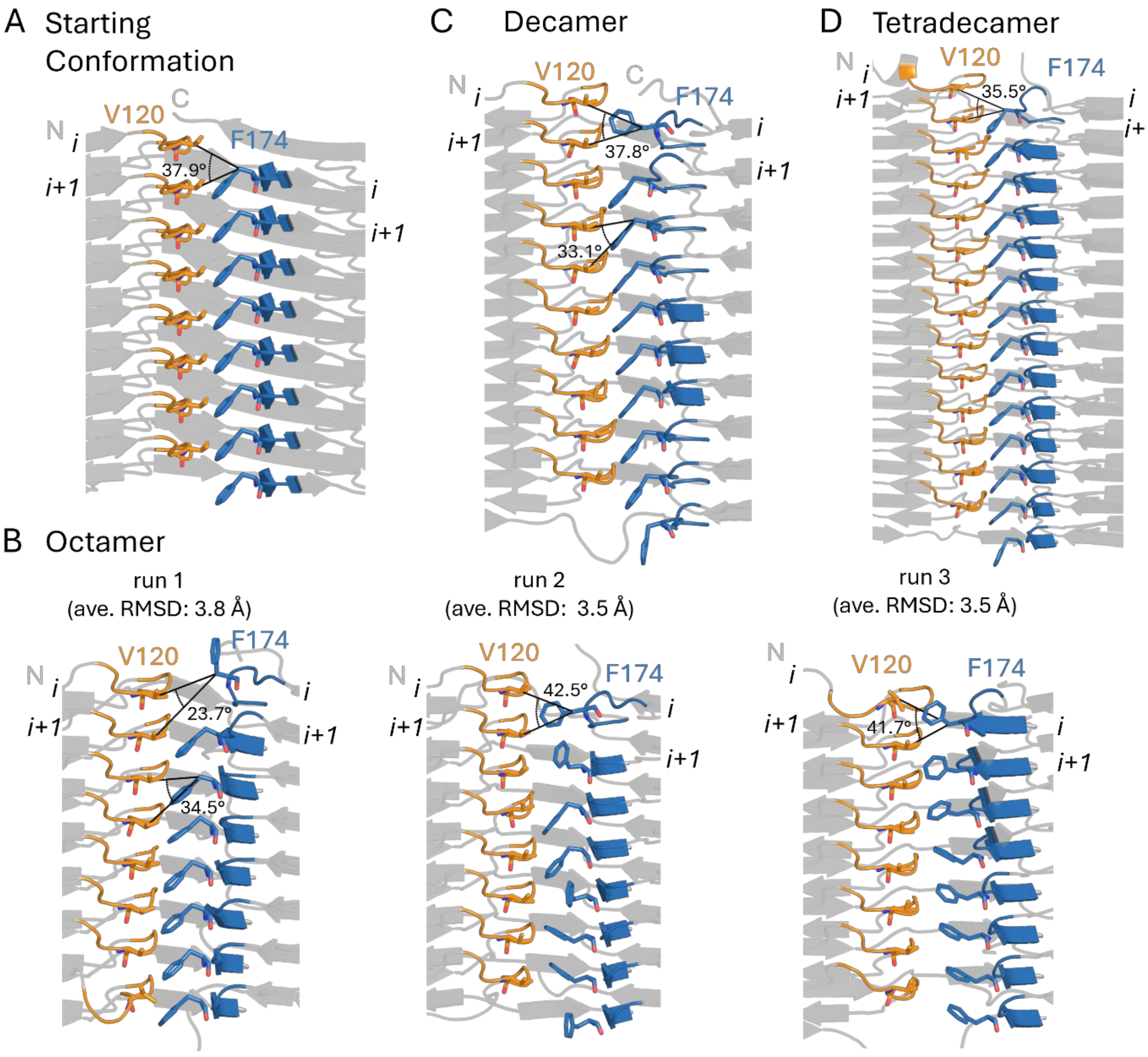

Figure S10. Degree of stagger measured by the angle between the CB of V120 and F174 of the same chain (*i*) and V120 of the subsequent chain (*i*+1). A) Stagger angle of starting conformation: 37.9°. **B)** Stagger angle of all three runs of the octamer and corresponding average RMSD: (run 1, 23.7°; run 2, 42.5°; run 3, 41.7°). **C)** Stagger angle of representative decamer run: 37.8° **D)** Stagger angle of representative tetradecamer run: 35.5°

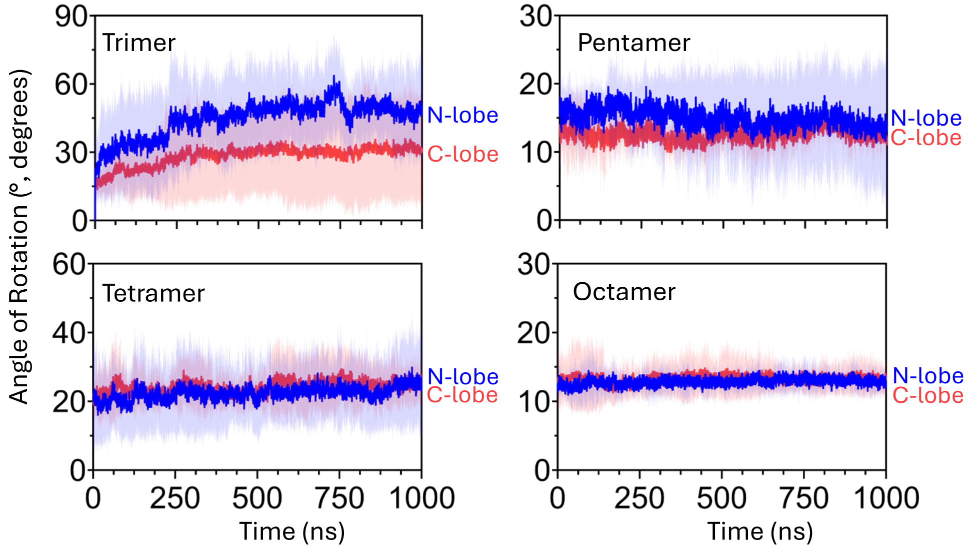

Figure S11. Degree of rotation of the N-and C-lobes. Average angle of rotation of the last 1000 ns for each multimer plotted as a function of time. The angle of rotation was determined with the coor orie functionality in CHARMM, by first aligning the initial fibril structure horizontally along the x-axis and rotating the center of mass of either the N- (blue) or C-lobes (red) of a subsequent frame to the x-axis.

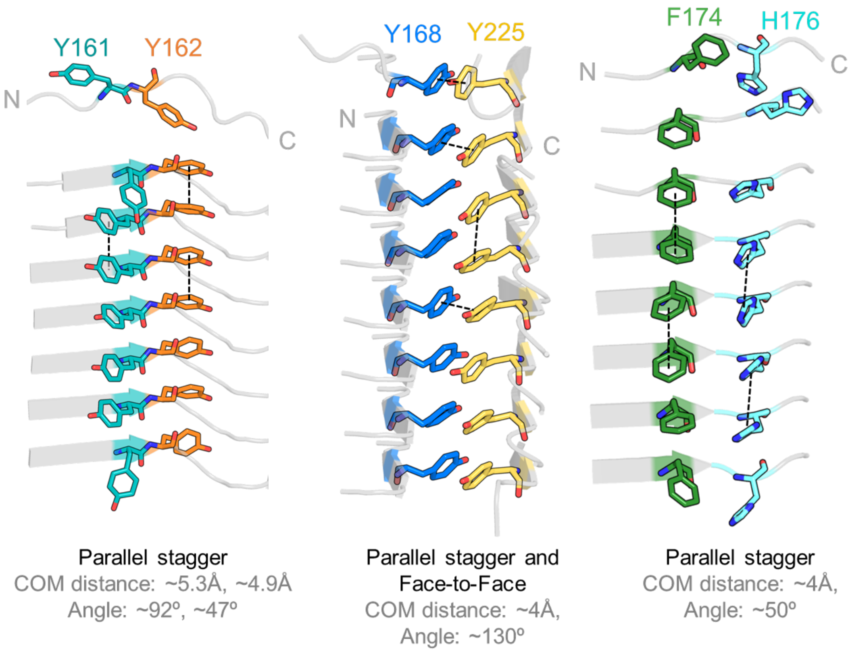

Figure S12. Determination of π-π interactions. Geometric parameters were used to approximate intra- and intermolecular π-π interactions of Y161:Y152, Y168:Y225, and F174:H176.

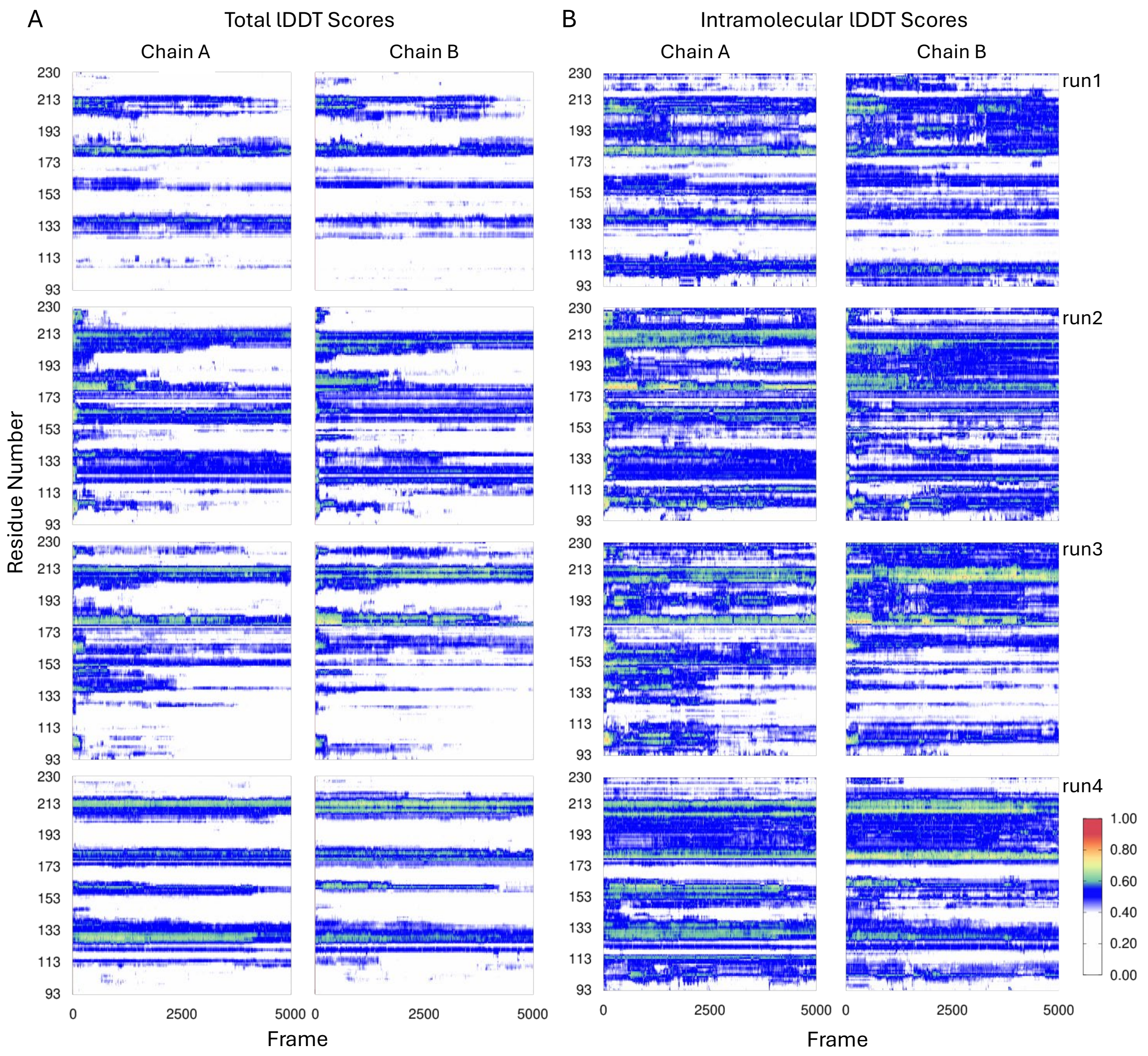

Figure S13. lDDT analysis of PrP^{Sc} dimer. A) Total lDDT scores for each residue of the dimer for the last 1 μs in simulation runs 1-4 plotted as a function of frame number. Each chain is displayed separately. **B)** Intramolecular lDDT scores for each residue of the dimer for the last 1 μs in simulation runs 1-4 plotted as a function of frame number. Each chain is displayed separately. Heatmap indicates lDDT scores from 0 to 1, where white represents scores below 0.4.

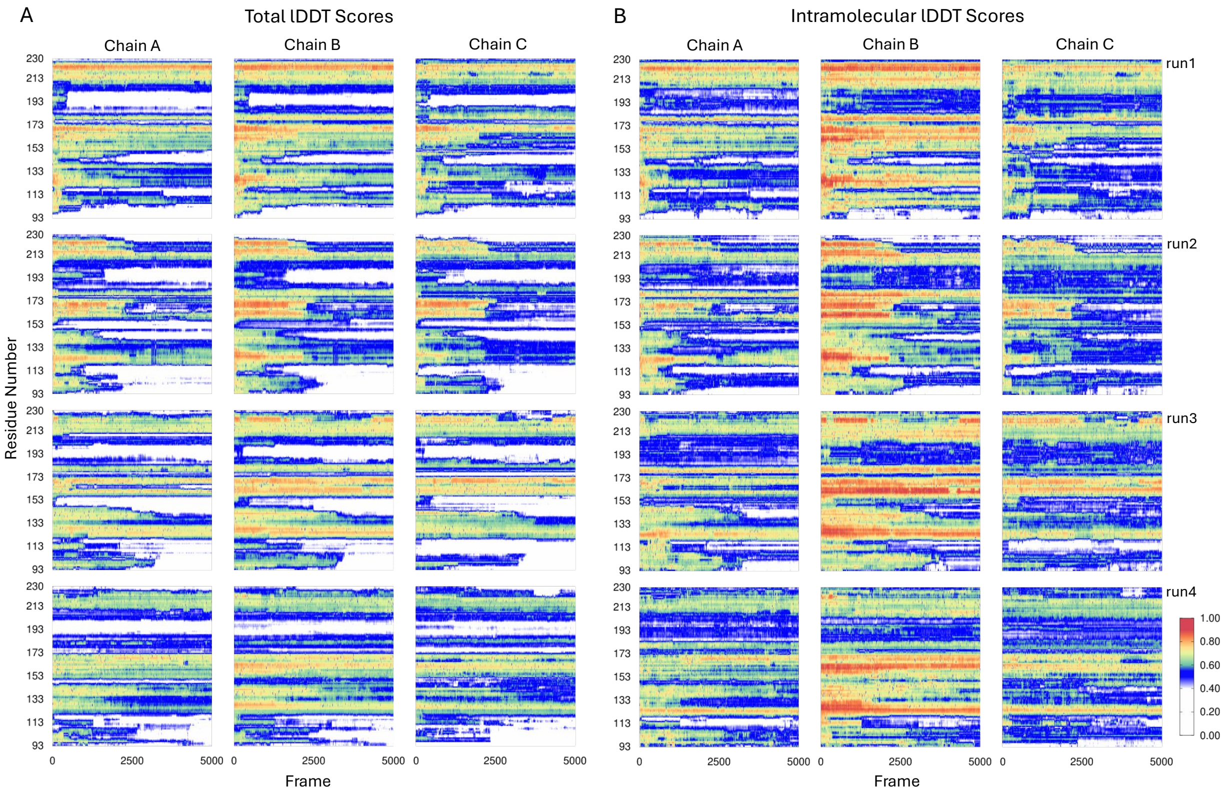

Figure S14. lDDT analysis of PrP^{Sc} trimer. A) Total lDDT scores for each residue of the trimer for the last 1 μs in simulation runs 1-4 plotted as a function of frame number. Each chain is displayed separately. **B)** Intramolecular lDDT scores for each residue of the trimer for the last 1 μs in simulation runs 1-4 plotted as a function of frame number. Each chain is displayed separately. Heatmap indicates lDDT scores from 0 to 1, where white represents scores below 0.4.

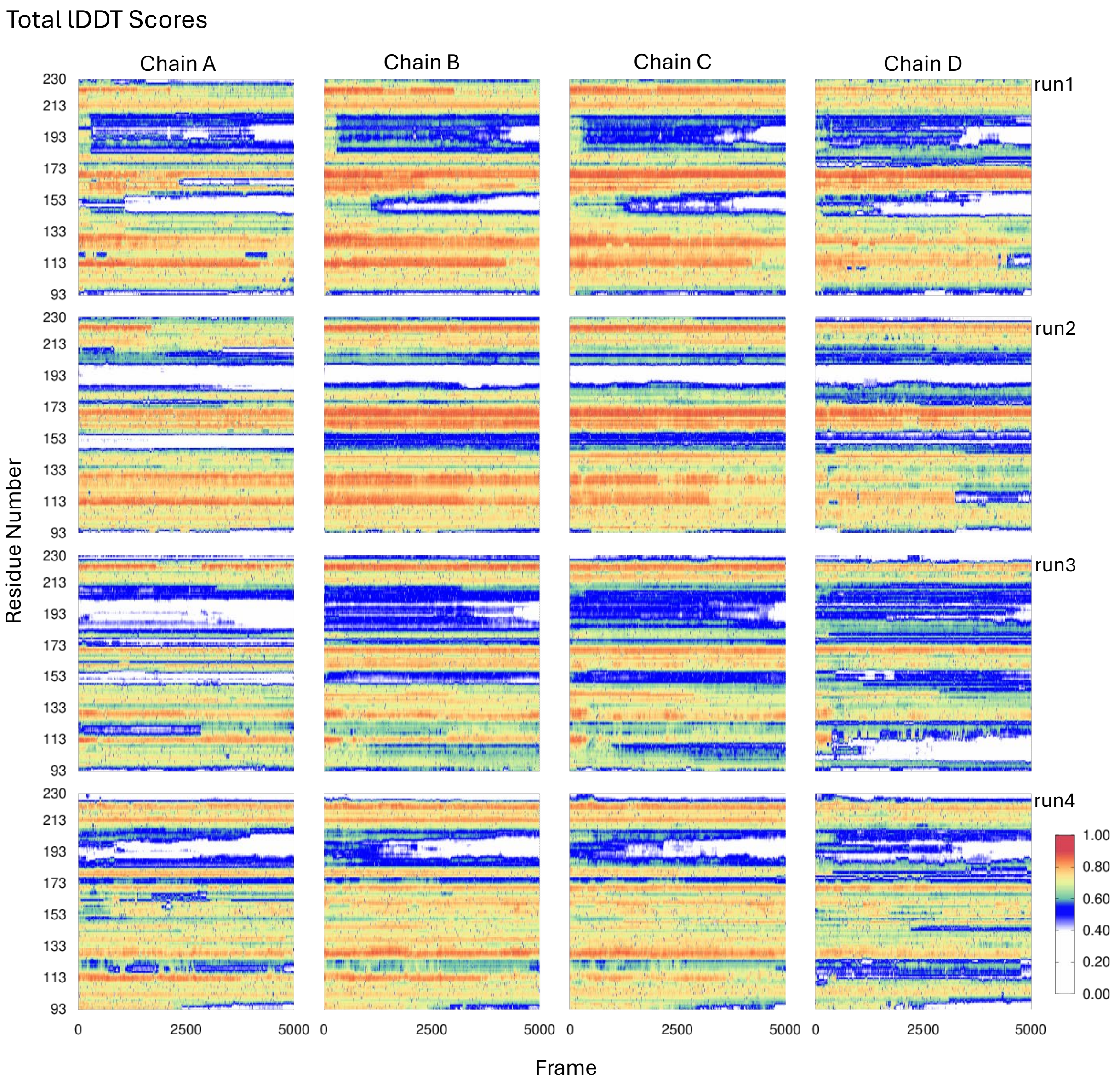

Figure S15. lDDT analysis of PrP^{Sc} tetramer. Total lDDT scores for each residue of the tetramer for the last 1 μs in simulation runs 1-4 plotted as a function of frame number. Each chain is displayed separately. Heatmap indicates lDDT scores from 0 to 1, where white represents scores below 0.4.

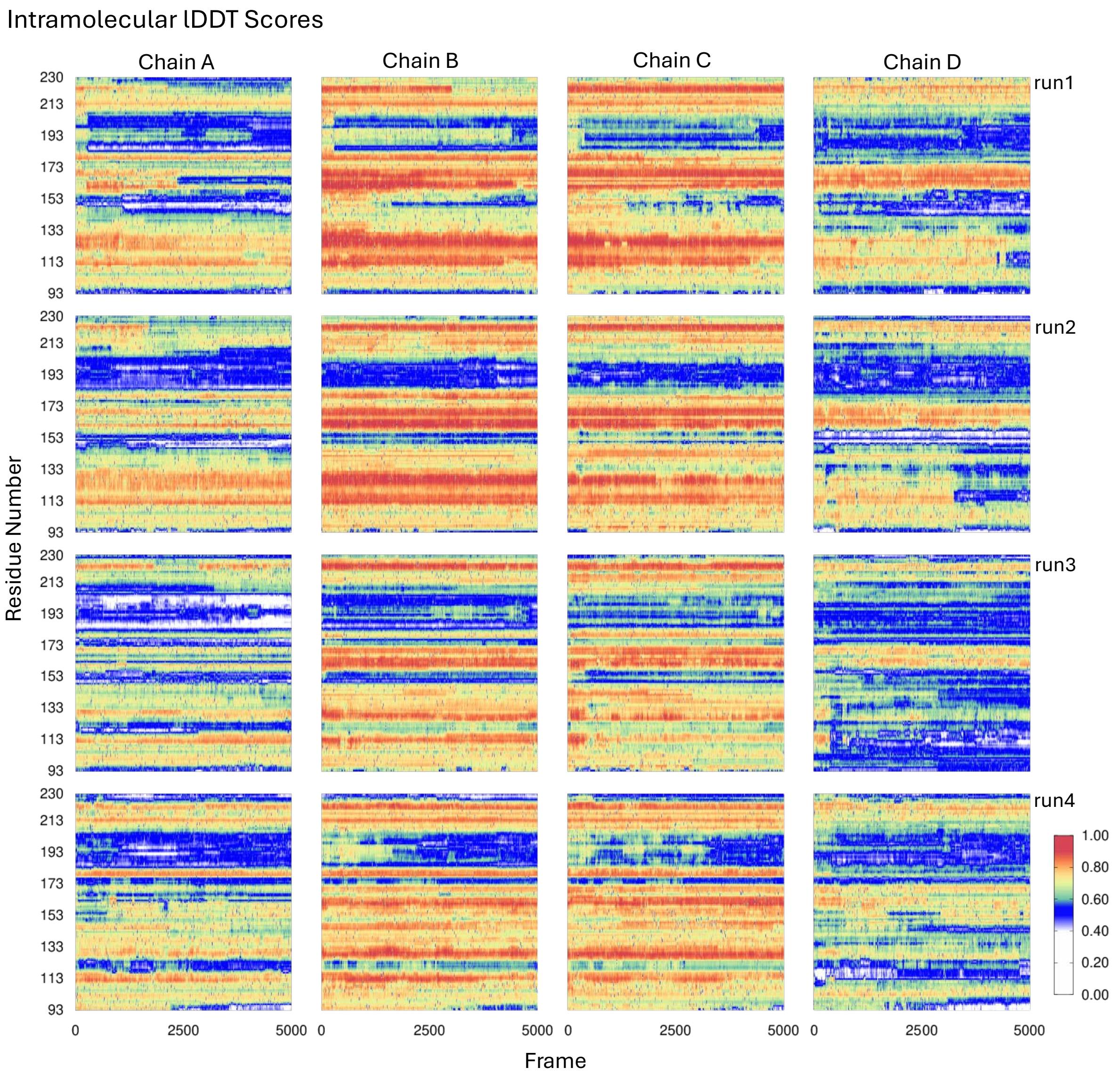

Figure S16. Intramolecular lDDT analysis of individual chains of PrP^{Sc} tetramer. Intramolecular lDDT scores for each residue of the tetramer for the last 1 μs in simulation runs 1-4 plotted as a function of frame number. Each chain is displayed separately. Heatmap indicates lDDT scores from 0 to 1, where white represents scores below 0.4.

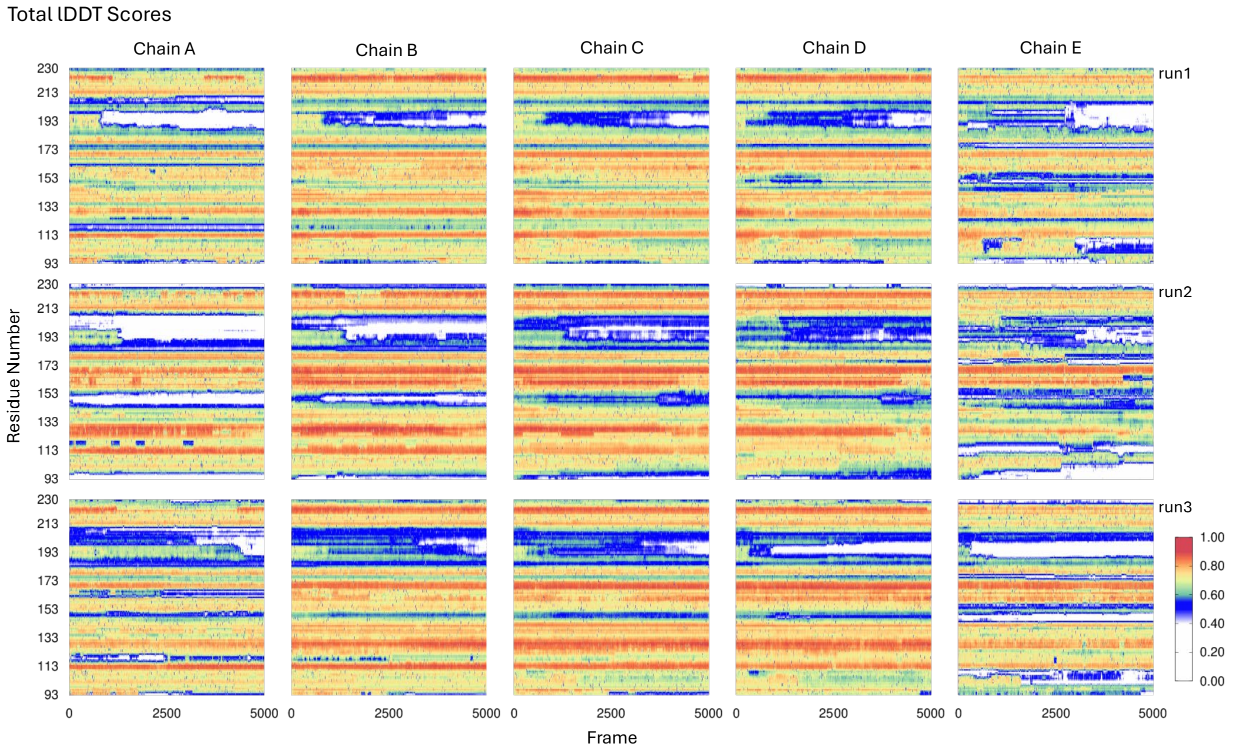

Figure S17. lDDT analysis of individual chains of PrP^{Sc} pentamer. Total lDDT scores for each residue of the pentamer for the last 1 μs in simulation runs 1-3 plotted as a function of frame number. Each chain is displayed separately. Heatmap indicates lDDT scores from 0 to 1, where white represents scores below 0.4.

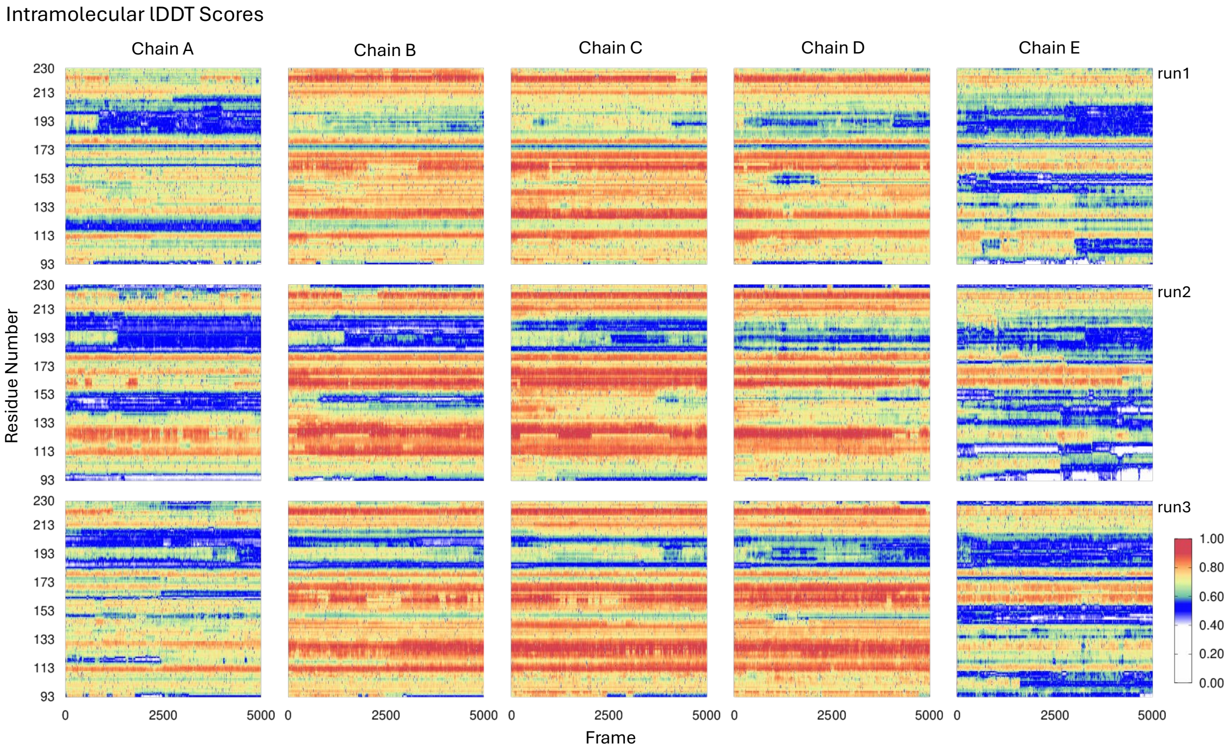

Figure S18. Intramolecular lDDT analysis of individual chains of PrP^{Sc} pentamer. Intramolecular lDDT scores for each residue of the pentamer for the last 1 μs in simulation runs 1-3 plotted as a function of frame number. Each chain is displayed separately. Heatmap indicates lDDT scores from 0 to 1, where white represents scores below 0.4.

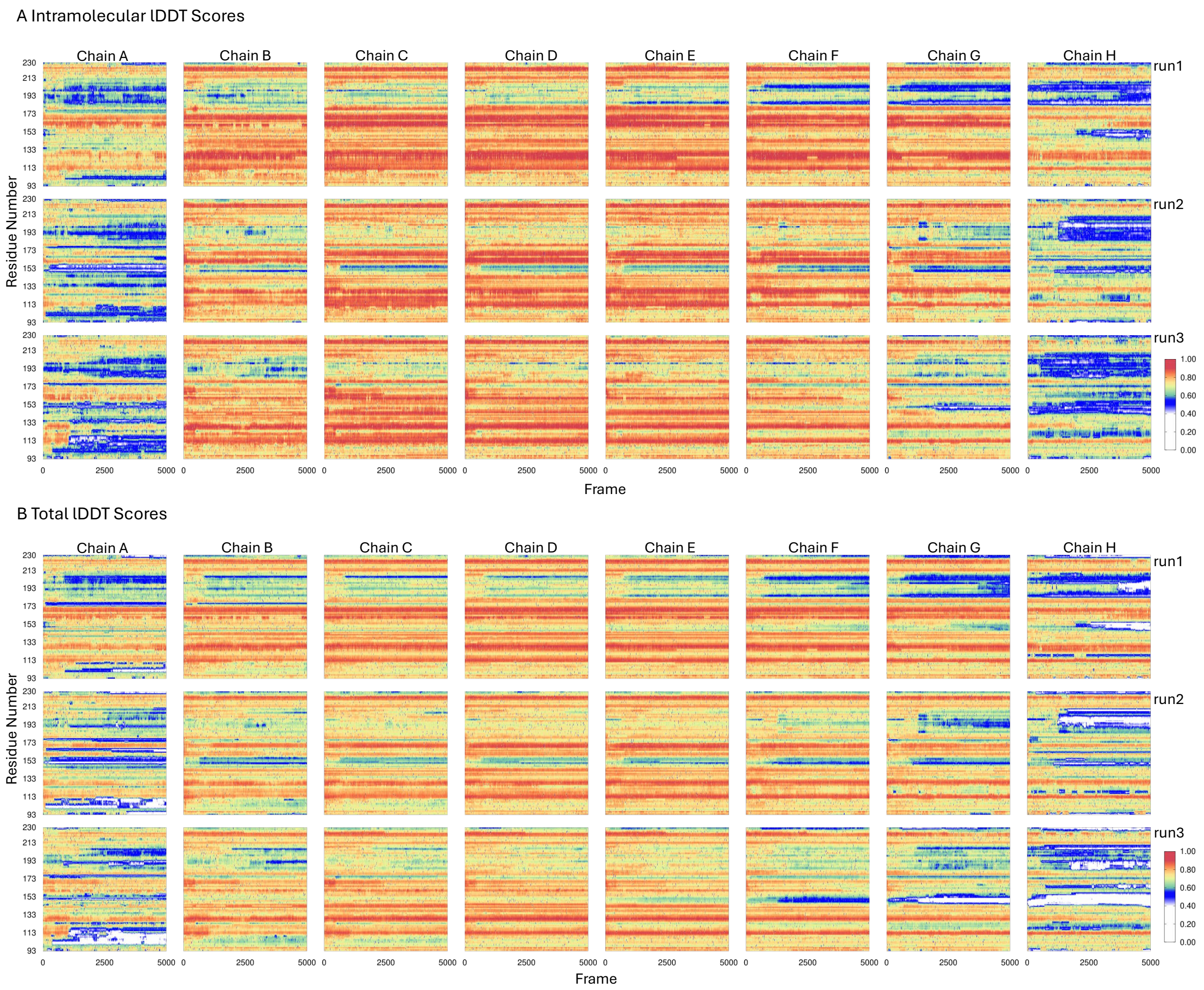

Figure S19. lDDT analysis of individual chains of PrP^{Sc} octamer. A) Intramolecular lDDT scores for each residue of the octamer for the last 1μs in simulation runs 1-3 plotted as a function of frame number. Each chain is displayed separately. **B)** Total lDDT scores for each residue of the octamer for the last 1 μs in simulation runs 1-3 plotted as a function of frame number. Each chain is displayed separately. Heatmap indicates lDDT scores from 0 to 1, where white represents scores below 0.4.

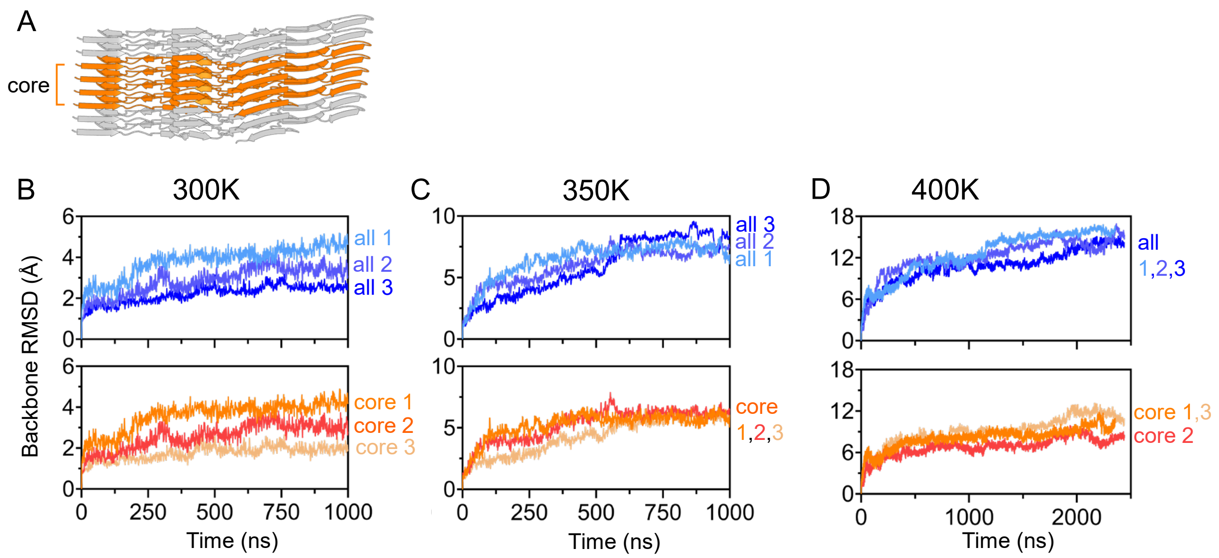

Figure S20. Temperature dependence of PrP^{Sc} octamer. A) Middle four chains of octamer comprising the core. The backbone RMSD of all chains (top panel) or core chains (bottom panel) for each MD run labeled 1-3 at **B)** 300K, **C)** 350K or **D)** 400K.

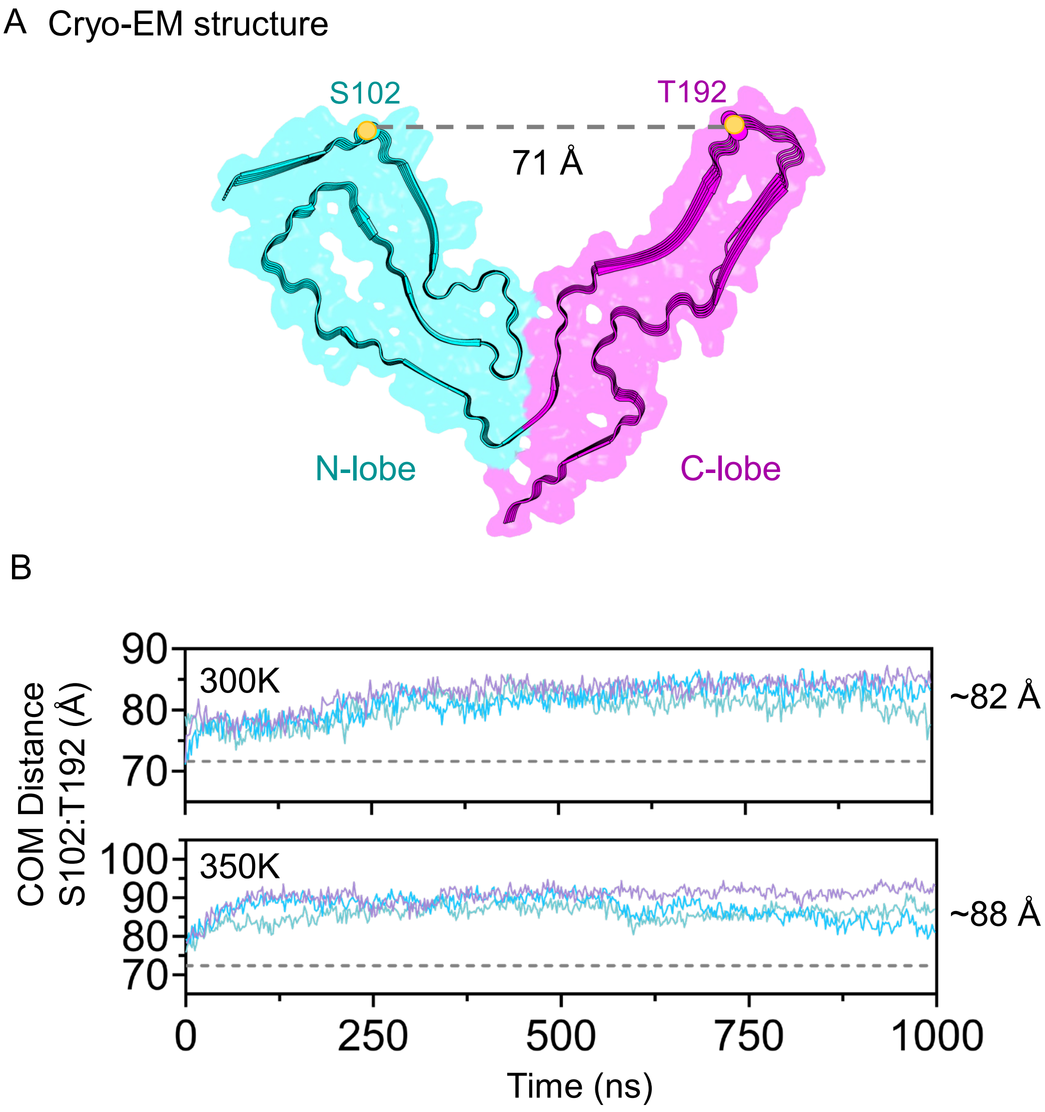

Figure S21. Temperature dependence of width of the cleft between the N- and C-lobes of octamer. Widths were evaluated by taking the center of mass (COM) for the two residues located at the tip of each lobe. Residues S102 and T192 were selected for the N- and C-lobes, respectively. The center of mass calculation included residues 102 or 192 on all the chains of the octamer. **A)** Cleft width of the cryo-EM structure. **B)** Time course of cleft width for three MD runs (run 1, purple; run 2, teal; run 3, cyan) at 300K and 350K. Distances are graphed at an interval of 2 ns for legibility and the dashed line is at 71 Å.

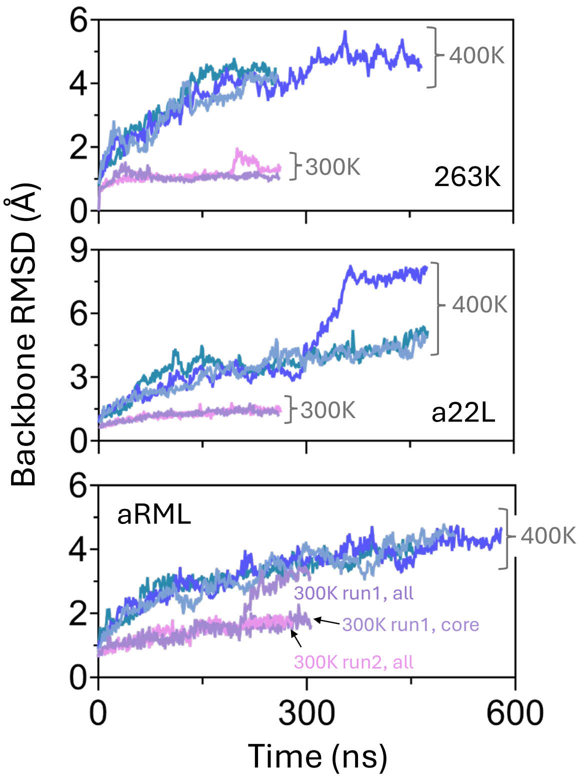

Figure S22. Backbone RMSD of 25-mer PrP^{Sc} Fibrils. Backbone RMSD computed for all chains or the core chains (as labeled) in reference to the first frame of each production run for simulations at either 300K and 400K for 263K PrP^{Sc} (top), a22L PrP^{Sc} (middle) or aRML PrP^{Sc} (bottom) 25-mers.
